# Multi-active phlorotannins boost antimicrobial peptide LL-37 to promote periodontal tissue regeneration in diabetic periodontitis

**DOI:** 10.1016/j.mtbio.2025.101535

**Published:** 2025-01-31

**Authors:** Cancan Li, Luowen Du, Yingying Xiao, Lei Fan, Quanli Li, Chris Ying Cao

**Affiliations:** a College & Hospital of Stomatology, Anhui Medical University, Key Lab. of Oral Diseases Research of Anhui Province, Hefei, 230032, China; b Institute of Oral Science, Department of Stomatology, Longgang Otorhinolaryngology Hospital of Shenzhen, Shenzhen, 518172, China

**Keywords:** Diabetic periodontitis, Phlorotannin, Antimicrobial peptide, Immune regulation, Periodontal tissue regeneration

## Abstract

The bidirectional correlation between diabetes and periodontitis positions the latter as the most prevalent complication of the former. Rehabilitation of the periodontal tissues damaged by diabetic periodontitis presents a significant clinical challenge. The multifaceted nature of the pathogenesis of diabetic periodontitis necessitates a comprehensive approach in its treatment to mitigate its adverse effects. To address this, a temperature-sensitive hydrogel containing phlorotannins (PL) and antimicrobial peptide LL-37 was developed to shift the microenvironment of diabetic periodontitis from an exacerbated high-glycemic inflammatory state to a regenerative one. The addition of PL significantly enhanced the antimicrobial properties, stability, and safety of LL-37. Vitro experiments confirmed that PL/LL-37 had good biocompatibility and promoted osteogenic differentiation of bone. PL/LL-37 demonstrated antioxidant properties by scavenging DPPH free radicals and inhibiting NO production. Furthermore, PL/LL-37 effectively modulated macrophage polarization from a M1 phenotype to an M2 phenotype through NF-κB P-p65 inflammatory pathway, thereby reducing the release of pro-inflammatory cytokines and promoting the secretion of anti-inflammatory cytokines. Interestingly, it could downregulate the AGE-RAGE signaling pathway, exerting a protective effect against diabetes. In addition, PL/LL-37 could attenuate inflammation levels, inhibit osteoclast production, promote bone regeneration, inhibit apoptosis and decrease RAGE levels in a rat model of diabetic periodontitis. These combined features synergistically accelerate diabetic periodontal bone regeneration. Consequently, PL/LL-37 emerges as a promising candidate for clinical treatment of diabetic periodontitis.

## Highlights

• PL has diverse biological activities, including antioxidant, anti-inflammatory, antimicrobial, and diabetic protection.
• LL-37 has potent antimicrobial activity, providing robust evidence for its potential application in periodontitis treatment.
• PL and LL-37 can form a temperature-responsive hydrogel, presenting an innovative approach for drug delivery and application.
• PL was first introduced as a therapeutic approach for diabetic periodontitis, providing a novel perspective for future study.

## Graphical abstract

Scheme 1 PL/LL-37 promotes periodontal tissue regeneration in diabetic periodontitis. Firstly, PL/LL-37 disrupts the *P. gingivalis* bacterial biofilm and binds endotoxin to exert an antibacterial effect. Secondly, PL/LL-37 down-regulates the NF-κB P-p65 pathway, promoting macrophage M1 to M2 conversion for enhanced anti-inflammatory effects. Thirdly, PL/LL-37 scavenges DPPH free radicals and down-regulates iNOS and NO production, further enhancing its anti-inflammatory properties. Additionally, PL/LL-37 reduces osteoclast production while upregulating RUNX2, BSP, and OPG production for improved osteogenesis. Moreover, it decreases Bax production while increasing Bcl-2 expression to prevent apoptosis. Finally, by reducing RAGE accumulation and inhibiting the AGE-RAGE reaction process, PL/LL-37 exhibits a protective effect against diabetes.

**Figure:**
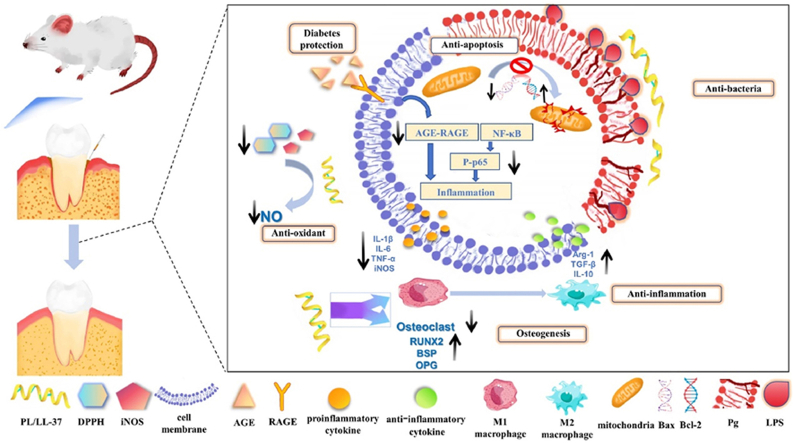

## 1 Introduction

Periodontal disease, an infectious and inflammatory condition affecting the periodontal tissues, is primarily caused by gram-negative anaerobic bacteria. It results in the degradation of tooth-supporting structures, including the periodontium and alveolar bone [1]. Furthermore, periodontitis is intricately associated with various systemic diseases, establishing a bidirectional relationship with diabetes [2]. Recognized as the sixth major complication of diabetes, periodontitis exhibits higher incidence and severity in diabetic patients compared to the general population, serving as a primary contributor to tooth loss in individuals with diabetes [3].

Currently, the treatment approach for diabetic periodontitis involves optimizing glycemic control in conjunction with comprehensive periodontal therapy [4], encompassing procedures such as periodontal curettage, antimicrobial therapy, and regenerative periodontal surgery like guided tissue regeneration. Nevertheless, the impact of antidiabetic medications on periodontal conditions remains uncertain. Moderate-certainty evidence suggests that periodontal treatment with subgingival appliances significantly enhances glycemic control in patients with periodontitis and diabetes, compared to no treatment or routine care [5]. Compared with subgingival scraping and root planning alone, patients with good glycemic control who also used topical antimicrobial agents showed greater reduction in periodontal probing depth (PD) and greater increase in clinical attachment level (CAL), especially in deep periodontal pockets [6]. However, it has been demonstrated that additional treatment involving topical and systemic antibiotics can temporarily induce antibiotic resistance in subgingival microorganisms [7]. Therefore, when prescribing systemic adjunctive antibiotics, careful consideration should be given to whether the clinical benefits of a slight increase in PD reduction outweigh the potential side effects.

It is worth noting that damage of periodontal tissue caused by diabetic periodontitis remains a challenging issue. Various biomaterials have been employed for periodontal bone regeneration, including growth-factor-loaded scaffolds [8], composite graded melt electro-written scaffolds [9], stem cell tablets [10], 3D printed multiphase scaffolds [11,12], and others. In addition, there have been studies on the construction of periodontal hard and soft tissue biomimetic interfaces [13], and these biomimetic systems have included nano-fiber scaffolds, cellular tablets, and hydrogels. These strategies primarily focus on promoting the differentiation of mesenchymal cells into odontoblasts and facilitating the formation of new osteoid fibers. Consequently, this leads to the attachment of new periodontal ligament fibers to the root and alveolar bone [14]. These strategies involve the development of organoids. Organoids are biomimetic tissue analogs generated through the self-organization of stem cells or adult cells, based on principles of developmental biology [15]. However, current organoids remain limited in terms of long-term culture, maturation, complexity, and functionality, primarily due to a lack of vascularization within these analogs [15]. Furthermore, the efficacy of these treatments in diabetic individuals is limited due to hyperglycemia. Functional cells such as fibroblasts and osteoblasts are more prone to apoptosis in patients with diabetic periodontitis, while osteoclast activity is heightened [16]. Consequently, diabetic patients experience significantly poorer postoperative outcomes compared to their non-diabetic counterparts due to insufficient bone formation, elevated secondary bone resorption and frequent reinfection [4].

The pathogenesis of diabetic periodontitis is believed to involve mechanisms such as inflammatory immune response [17], accumulation of advanced glycosylation end-products (AGEs) [18], oxidative stress, apoptosis [19], and insulin resistance (IR) [20], which synergistically contribute to the disease progression. Elevated levels of glucose, reactive oxygen species (ROS), and AGEs have been detected in the periodontal tissues of diabetic patients. This heightened presence leads to increased activation of nuclear factor-κB (NF-κB) and subsequent expression of inflammatory cytokines, including tumor necrosis factor (TNF) and interleukin (IL-1, IL-6) [21]. TNF-α and IL-6 have been shown to disrupt intracellular insulin signaling, potentially leading to IR [22,23]. Furthermore, *porphyromonas gingivalis* (*P. gingivalis*) triggers a systemic inflammatory response that impairs insulin signaling pathways, induces pancreatic β-cell hypoplasia, and ultimately results in decreased insulin sensitivity and IR [24]. Hyperglycemia further exacerbates inflammation, oxidative stress, and apoptosis, contributing to the progression of diabetic complications [25]. Additionally, AGEs negatively impact bone metabolism by impairing bone repair and formation [26], and reducing extracellular matrix production [27].

Seaweed extracts and its bioactive compounds have antidiabetic potential [28]. As a kind of seaweed extract, the phlorotannins (PL) exhibits a diverse array of biological activities, encompassing antioxidant [29], anti-inflammatory [30], antibacterial [31,32], antitumor [29], matrix metalloproteinases inhibition [33], anti-cardiovascular disease effects, as well as antidiabetic effects involving the inhibition of amylase and glucosidase, and reduction of AGEs levels in tissues [[34], [35], [36], [37], [38]]. Furthermore, PL demonstrates hepatoprotective properties and inhibits hyaluronidase activity while also exerting lysozyme-like actions [39]. Notably, it significantly attenuates the secretion of inflammatory cytokines (TNF-a and IL-6) in lipopolysaccharide (LPS) -stimulated macrophages [40]. It also possesses antioxidant properties that reduce LPS-induced lipid peroxidation in gingival epithelial cells [40]. Taking the advantage of these properties, PL can be considered for the anti-inflammatory, antioxidative stress, and osteogenetic requirements of diabetic periodontitis. However, it is worth noting that research suggests PL may not be effective against the growth and/or biofilm formation of Streptococcus pyogenes and *P. gingivalis* [40]. Therefore, there is an urgent need to introduce a drug that can efficiently inhibit periodontal pathogens.

LL-37 is derived from hCAP-18, the only human histatin antimicrobial peptide currently discovered that is sensitive to the pathogenic bacteria of periodontitis, such as *P. gingivalis* [41], *Aggregator actinobacillus* [42], *Prevotella intermedia*, and *Clostridium nucleatum* [43], all of which are causative agents of periodontitis. The mechanism of action for LL-37 involves the formation of ion channels that inhibit and destroy bacterial cell membranes. Furthermore, LL-37 rapidly binds to LPS, an endotoxin present on the surface of bacterial membranes, effectively neutralizing bacteria [44]. Additionally, LL-37 exhibits anti-biofilm effects through various mechanisms including inhibition of bacterial adhesion, down-regulation of biofilm-associated genes, interference with population-sensing pathways, degradation of biofilm matrix components and eradication of biofilm-resident cells [45]. In this study, we synthesized a temperature-sensitive hydrogel of PL/LL-37 by combining the multifunctional properties of PL and the antimicrobial properties of LL-37. *In vitro* studies have demonstrated that PL/LL-37 exhibits antimicrobial, antioxidant, anti-inflammatory, anti-diabetic, and bone-enhancing properties. Furthermore, animal experiments have validated its efficacy in reducing inflammation, promoting periodontal bone regeneration in diabetic conditions, providing protection against diabetes-related complications, and exhibiting anti-apoptotic effects. These findings present a novel therapeutic option for the management of diabetic periodontitis.

## 2 Materials and methods

### 2.1 Synthesis and evaluation of PL/LL-37

#### 2.1.1 Determination of total phenolic content in PL

The PL powder with a purity of 98 % was procured from Xi'an Qiannuo Biotechnology Co., LTD. The determination of total phenol content in PL was performed using the Folin-Ciocalteu (F-C) method [46]. Briefly, 20 μL PL, 10 μL F-C reagent and 130 μL of distilled water were combined and mixed thoroughly. The solution was incubated for 5 min at room temperature in darkness. Subsequently, 40 μL of 20 % Na_2_CO_3_ was added to the mixture, followed by thorough mixing and further incubation for 1 h at room temperature in darkness. Finally, the absorbance of the resulting solution was measured at a wavelength of 620 nm (SHIMADZU, UV-1800, China). Distilled water served as a blank control, while phloroglucinol was used to construct the standard curve. The total phenol content in PL was expressed as μg × mg^−1^ of phloroglucinol equivalent.

#### 2.1.2 Synthesis of LL-37 and PL/LL-37

The LL-37 (LLGDFFRKSKEKIGKEFKRIVQRIKDFLRNLVPRTES) was synthesized using the standard 9-fluorenylmethoxycarbonyl solid-phase method by Shanghai Science Peptide Biological Technology Co. LTD. The synthetic LL-37 peptide was purified and characterized through reversed-phase high-performance liquid chromatography (HPLC) and mass spectrometry (MS). A solution of LL-37 was prepared in phosphate-buffered saline (PBS) buffer at a final concentration of 2 mg/mL.

The PL solution was prepared in PBS buffer and mixed with the LL-37 solution at various ratios to form different concentrations of PL/LL-37 (10 mg/mL PL, 50, 100, 150, 200, 250, and 300 μg/mL LL-37). The secondary structures of LL-37 (100 μg/mL) and PL/LL-37 (10 mg/mL PL, 100 μg/mL LL-37) were analyzed using a circular dichroism (CD) spectrometer (JASCO, Japan) within the wavelength range of 190–260 nm.

#### 2.1.3 Cytocompatibility test

We used MC3T3-E1 cells, Rat Bone Mesenchymal Stem Cells (BMSCs) and mouse macrophages RAW264.7 to assess cytocompatibility. MC3T3-E1 cells were cultured in α-MEM (Gibco, Grand Island, USA) supplemented with 10 % fetal bovine serum (Gibco, Grand Island, USA) and 1 % penicillin-streptomycin (Gibco, Grand Island, USA) at a temperature of 37 °C and a CO_2_ concentration of 5 %. Once the cells reached a confluence of 70–80 %, they were detached using 0.25 % trypsin (Gibco, Grand Island, USA) and passaged for subsequent experiments. SPF grade three-week-old male Sprague-Dawley (SD) rats, weighing approximately 40 g, were selected for this experiment with the approval of the Medical Ethics Committee of Anhui Medical University (Approval No. LLSC20232193). BMSCs were harvested from both the femur and tibia and cultured in high-glucose DMEM medium (with 4.5 g/L D-glucose, Gibco, Germany) supplemented with 10 % fetal bovine serum (Gibco, Grand Island, USA). The cultures were maintained in a cell culture incubator (Thermo Fisher, USA) at 37 °C with 5 % CO_2_ and sub-cultured when confluence reached 70−80 %. The third-generation BMSCs were chosen for subsequent experiments due to their superior purity and viability. The RAW264.7 were cultured in high-glucose DMEM medium (with 4.5 g/L D-glucose, Gibco, Grand Island, USA) supplemented with 10 % fetal bovine serum (Every green, Zhejiang, China) at 5 % CO_2_ and 37 °C. Once the cells reached a confluence of 70–80 %, they were passaged for subsequent experiments.

The cells were seeded into 96-well plates with 5 × 10^3^ cells per well and incubated with varying concentrations of PL (10 mg/mL, 20 mg/mL), LL-37 (50, 100, 150, 200, 250, and 300 μg/mL) and PL/LL-37 (10 mg/mL PL, 50, 100, 150, 200, 250, and 300 μg/mL LL-37) for durations of 1 day, 3 days and 5 days. The control group was treated with PBS, while the positive control groups were treated with 0.12 % chlorhexidine (CHX) and 25 μM minocycline (MC), respectively. Cell proliferation was assessed using the cell counting kit-8 (CCK-8) method (Biosharp, Beijing, China) with absorbance measured at 450 nm (Bio-tek, MQX200, USA) after a 2 h incubation.

### 2.2 Evaluation of the antioxidant properties of PL, LL-37 and PL/LL-37

#### 2.2.1 2,2-Diphenyl-1-(2,4,6-trinitrophenyl) hydrazyl (DPPH) assay

The antioxidant properties of PL/LL-37 were assessed according to the instructions of the total antioxidant capacity assay kit (McLean, Shanghai, China). As per the kit guidelines, absorbance values at 515 nm (SHIMADZU, UV-1800, China) were measured for the blank group, PL, LL-37, and PL/LL-37 group. Each group's absorbance was recorded thrice and used to calculate and statistically analyze the DPPH free radical clearance rate (%).

#### 2.2.2 Determination of total intracellular nitric oxide (NO) content

The RAW264.7 cells were seeded in a 24-well plate with a density of 1 × 10^5^ cells per well. After incubation for 24 h, the cells adhered to the wells. The medium was replaced in the control group and LPS group, while the experimental group received additional treatment and was further incubated for 24 h. Subsequently, both the LPS group and the experimental group were stimulated *P. gingivalis*-LPS for 4 h. The total intracellular NO content of each group was evaluated using the Total Nitric Oxide Assay Kit (Beyotime, Shanghai, China) and OD values were measured at the absorbance of 550 nm using a microplate reader (Bio-tek, MQX200, USA).

### 2.3 Antibacterial ability of PL, LL-37 and PL/LL-37

The *P. gingivalis* (ATCC33277) strain was cultured in Brain Heart Infusion Broth (BHI, Hopebio Qingdao, Qingdao, China) under anaerobic conditions (85 % N_2_, 10 % H_2_, 5 % CO_2_) at 37 °C. After centrifugation at 4000 rpm for 10 min, the bacteria were resuspended in BHI medium and plated on Columbia blood agar plates. Prior to each experiment, a single colony was selected and cultivated for 48 h to establish a bacterial stock solution. The concentration of bacteria was diluted to 1 × 10^6^ CFU ml^−1^ for the subsequent experiments using a microplate reader at the absorbance of 600 nm.

#### 2.3.1 Disc diffusion

To evaluated the bacteriostatic activity, circular filter paper pieces with a diameter of 6 mm were saturated with 20 μL liquid of 10 mg/mL PL, 100 μg/mL LL-37, PL/LL-37 (10 mg/mL PL, 50, 100, 150, 200, 250, and 300 μg/mL LL-37), CHX and PBS, respectively. Subsequently, these impregnated filter paper pieces were placed onto blood agar plates that had been previously coated with *P. gingivalis* bacterial stock solution. The plates were then incubated at 37 °C. After 7 days incubation, the bacteriostatic activity exhibited by each sample was assessed by measuring the zone of inhibition in millimeters.

#### 2.3.2 The ability to resist planktonic bacteria

The PL, LL-37, and PL/LL-37 were co-cultured with *P. gingivalis* at a volume ratio of 1:1, respectively. Meanwhile, CHX and MC were used as positive control groups and PBS was used as a negative control group. After co-cultivation for 4 h, the liquid in the 96-well plate was diluted to 10^−3^ times. Then, 10 μL of *P. gingivalis* bacterial suspension from each well were extracted and plated on BHI agar plates individually followed by incubation at 37 °C for 48 h. Subsequently, the colonies were enumerated, and the inhibition rate was calculated and subjected to statistical analysis. The formula used for calculating the bacterial inhibition rate is as follows: [(number of colonies in the Pg group - number of colonies in the experimental group)/number of colonies in the Pg group] ∗ 100 %.

#### 2.3.3 Inhibition of *P. gingivalis* growth

The aforementioned PL, LL-37, and PL/LL-37 were co-cultured with *P. gingivalis* at a 1:1 vol ratio, respectively. Meanwhile, CHX and MC were used as positive control groups, while PBS was used as a negative control group. Subsequently, the OD_600_ value was measured during the 1–7day co-cultivation period using an enzyme marker to generate the growth curve of *P. gingivalis*.

#### 2.3.4 Inhibition of *P. gingivalis* adhesion and biofilm formation

##### 2.3.4.1 Scanning electron microscope (SEM) and Live/Dead staining

The cell slides were placed at the bottom of a 24-well plate. PL (20 mg/mL), LL-37 (200 μg/mL), PL/LL-37 (20 mg/mL PL, 200 μg/mL LL-37) diluent, 0.24 % CHX (positive control group), 50 μM MC (positive control group), and PBS (negative control group) were added to the respective wells of the 24-well plate. Each well was filled with 1 mL of suspension. After incubating for 24 h, *P. gingivalis* bacterial suspension with a final concentration of 1 × 10^6^ CFU mL^−1^ was added to each well of the 24-well plate (1 mL/well) and further incubated for 6 h. Some of the slides were initially fixed with 2.5 % glutaraldehyde for 4 h at 4 °C. Subsequently, they underwent a series of ethanol dehydration steps and critical point drying to eliminate any remaining moisture. Afterwards, they were examined under SEM (ZEISS Gemini SEM 300, Germany) to evaluate the inhibitory effects of drugs on bacterial adhesion and biofilm formation. The remaining slides were treated with the Live/Dead BacLight bacterial viability detection kit (Bestbio, China) and incubated in darkness for 30 min, and then the bacteria on the slides were observed using Standing fluorescence microscope (Leica, Germany).

##### 2.3.4.2 Crystal violet staining

A 100 μL suspension of PL (10 mg/mL), LL-37 (100 μg/mL), and PL/LL-37 (10 mg/mL PL, 100 μg/mL LL-37) was added to the well of the 96-well plate. PBS was used as the negative control group, with 5 replicates per set. After incubating for 72 h, a final concentration of *P. gingivalis* bacteria at 2 × 10^6^ CFU mL^−1^ was added to the 96-well plate (100 μL/well). Following another incubation period of 72 h, the treated plate was washed once with sterile double-distilled water (DDW), fixed by adding 100 μL of 4 % paraformaldehyde per well for 20 min at room temperature, and air-dried for an additional 20 min after washing with sterile DDW. Subsequently, 1 % crystal violet solution (Biosharp, Beijing, China) was used for staining for 20 min. After being washed again with sterile DDW, decolorization was achieved by treating the wells with anhydrous ethanol (100 μL/well). Finally, the OD_590_ value was measured using a microplate reader (Bio-tek, MQX200, USA) to evaluate the inhibitory effect on bacterial adhesion and biofilm formation.

#### 2.3.5 Anti-biofilm ability

##### 2.3.5.1 SEM and Live/Dead staining

The cell slides were placed at the bottom of a 24-well plate. A final concentration of 1 × 10^6^ CFU mL^−1^ of *P. gingivalis* bacteria was added to each well of 1 mL of suspension in the 24-well plate. After incubation for 24 h, the supernatant was discarded and the plate was washed once with sterile DDW. Subsequently, 1 mL suspension of PL (10 mg/mL), LL-37 (100 μg/mL), PL/LL-37 (10 mg/mL PL, 100 μg/mL LL-37) was added to the well of the 24-well plate. CHX and MC were used as the positive control groups, PBS was used as the negative control group. After culturing for 24 h, the 24-well plate was washed twice with sterile DDW before further analysis using SEM observation on some slides and Live/Dead staining followed by confocal laser scanning microscopy (CLSM, LSM880+airyscan, Germany) examination on other slides.

##### 2.3.5.2 Crystal violet staining

A final concentration of 1 × 10^6^ CFU mL^−1^ of *P. gingivalis* bacteria was added to a 96-well plate (200 μL/well). After incubation for 72 h, the *P. gingivalis* biofilms were formed at the bottom of the 96-well plates. Then the supernatant was discarded and the plate was washed once with sterile DDW. Subsequently, 200 μL suspension of PL (10 mg/mL), LL-37 (100 μg/mL), PL/LL-37 (10 mg/mL PL, 100 μg/mL LL-37) was added to the well of the plate. PBS was used as the negative control group, with 5 replicates per set. After culturing for 72 h, the wells were cleaned with sterile DDW and fixed by adding 100 μL of 4 % paraformaldehyde per well for 20 min at room temperature, and air-dried for an additional 20 min after washing with sterile DDW. Subsequently, 1 % crystal violet solution (Biosharp, Beijing, China) was used for staining for 20 min. After being washed again with sterile DDW, decolorization was achieved by treating the wells with anhydrous ethanol (100 μL/well). Then the OD590 value was measured using a microplate reader to evaluate the destruction effect on the formed *P. gingivalis* biofilms.

##### 2.3.5.3 BCA assay

In addition, BCA assay was used to assess bacterial lysis. Eighteen EP tubes were prepared. Then, 1 × 10^8^ CFU Pg bacteria were added to each tube and adjusted to a final volume of 2 mL with BHI. The tubes were randomly divided into six groups: the Pg group, PL group, LL-37 group, PL/LL-37 group, CHX group, and MC group, with three replicates for each condition. For the drug treatment groups, 2 mL of the respective drug solution was added; conversely, for the Pg control group, 2 mL of PBS was used.

Subsequently, the Pg control group underwent centrifugation at 12,000 rpm for 10 min. The supernatant obtained from this centrifugation was processed according to the instructions provided in the BCA kit to determine protein content. In contrast, samples from the drug treatment groups required an incubation period of 2 h prior to centrifugation for protein measurement. Finally, statistical analysis was performed on the protein content across all experimental groups.

#### 2.3.6 Effects of PL/LL-37 on morphology of *P. gingivalis* bacteria

The cell slides were placed at the bottom of a 24-well plate. PL (10 mg/mL), LL-37 (100 μg/mL), and PL/LL-37 (10 mg/mL PL, 100 μg/mL LL-37) were co-cultured with *P. gingivalis* in a volume ratio of 1:1, respectively. After 24 h, the cell slides were observed under SEM.

### 2.4 Evaluation of immunomodulation, diabetes protection, and osteogenic properties in vitro

Referring to several pieces of literature [[47], [48], [49]], we established an in vitro model of diabetic periodontitis using the following methods. We created this model by culturing RAW264.7 cells in a high-glucose environment (with 4.5 g/L D-glucose), and stimulating them with *P. gingivalis-*LPS (1 μg/mL) to replicate the inflammatory microenvironment associated with high glucose levels in diabetic periodontitis.

Firstly, RAW264.7 cells were cultured in high-glucose DMEM medium (Gibco, Grand Island, USA) supplemented with 10 % fetal bovine serum (Per Green, Zhejiang, China) under conditions of 5 % CO_2_ at 37 °C. Once the cells reached 70–80 % confluence, they were passaged for subsequent experiments. Following this step, RAW264.7 cells were stimulated with 1 μg/ml of gingival abscess-LPS to induce their transformation into M1-type macrophages during the experimental procedures, thereby constructing a hyperglycemic inflammatory microenvironment.

#### 2.4.1 Quantitative real-time PCR (qRT PCR)

In qRT PCR experiments, 1 × 10^6^ RAW264.7 cells were inoculated per well in 6-well plates and cultured for 24 h. Then, two experimental protocols were designed to verify the effect of PL/LL-37 on the level of cytokine mRNA secretion within RAW264.7 cells. In the first 6-well plate, the cells were treated with PL (10 mg/mL), LL-37 (100 μg/mL), and PL/LL-37 (10 mg/mL PL, 100 μg/mL LL-37) for 4 h and then stimulated with *P. gingivalis*-LPS at a concentration of 1 μg/mL for an additional 24 h. In the second 6-well plate, the cells were first stimulated with *P. gingivalis*-LPS at a concentration of 1 μg/mL for 24 h to polarize them into M1 morphology and then incubated with PL (10 mg/mL), LL-37 (100 μg/mL), and PL/LL-37 (10 mg/mL PL, 100 μg/mL LL-37) for 4 h.

Then the mRNA was extracted according to the instructions of the RNA-Quick Purification Kit (EScience Biotech, Shanghai, China) and its concentration was measured. Subsequently, the reverse transcription system was prepared according to the instructions, and carried out by shaking and shortening: 15 min at 37 °C, 5 s at 85 °C, and then transferred to 4 °C. The obtained sample was supplemented with 90 μL of DEPC water, mixed thoroughly by blowing and agitation, and stored at −80 °C. For the up-sampling system preparation, the Prime Script® RT reagent kit with gDNA Eraser (Takara, Kusatsu, Japan) protocol was followed. The prepared system was then added to an octuple tube which was tightly capped. Prior to flicking the bottom of the tube to remove air bubbles ensuring proper mixing of the system, centrifugation at 2000 rpm for a few seconds on the octuple tube was performed. The target fragment was subsequently amplified using the Light Cycler 96 qRT PCR System (Mx3000P, Agilent). The primer sequences were obtained from Sangon Biotech (Shanghai, China) and are provided in Table S1.

#### 2.4.2 Western blot (WB) assay

In WB experiments, 1 × 10^6^ RAW264.7 cells were inoculated per well in 6-well plates and cultured for 24 h. Then, the cells were first stimulated with *P. gingivalis*-LPS at a concentration of 1 μg/mL for 24 h to polarize them into M1 morphology and then incubated with PL (10 mg/mL), LL-37 (100 μg/mL), and PL/LL-37 (10 mg/mL PL, 100 μg/mL LL-37) for 24 h. The RAW264.7 cells were lysed using Radio-Immunoprecipitation Assay (RIPA) buffer (Beyotime, Shanghai, China) for 30 min to extract total proteins. The supernatant was collected by centrifugating at 12,000 g for 15 min at 4 °C. The protein concentrations were determined using a bicinchoninic acid protein assay kit (Beyotime, Shanghai, China). Subsequently, each protein sample (15 μg) was electrophoresed on a 10 % sodium dodecyl sulfate–polyacrylamide gel (Epizyme Biotech, Shanghai, China), followed by transfer onto a polyvinylidene fluoride membrane (Biosharp, Beijing, China). The membrane was blocked with 5 % skimmed milk at 37 °C for 2 h, followed by washing and overnight incubation at 4 °C with primary antibodies against the following proteins: NF-κB p65 (p65, 1:1000, Cell Signaling Technology), phosphorylated NF-κB p65 (P-p65, 1:1000, Cell Signaling Technology), Receptor for Advanced Glycation End Products (RAGE, 1:1000, Abcam), TNF-α (1:1000, Abcam), IL-1β (1:1000, Abcam), IL-6 (1:1000, Abcam) and β-actin (1:10,000, Zhongshan, Beijing, China). Subsequently, the membrane was washed five times with TBST buffer for 8 min each time and then incubated with horseradish peroxidase-conjugated IgG antibody for 2 h at 37 °C. HRP chemiluminescence substrate (Millipore, USA) was utilized according to the manufacturer's instructions to perform chemiluminescence detection. The intensity of the bands was quantified using a chemiluminescence western blot detection system. At least three samples from each group were analyzed in triplicate, and the blots were assayed using IMAGE J (National Institute of Health, Bethesda, MD, USA).

#### 2.4.3 Effect of PL/LL-37 on osteogenic differentiation of BMSCs

##### 2.4.3.1 Alkaline phosphatase (ALP) and alizarin red S (ARS) staining

The experiment was structured into five subgroups: NC group, LPS group, LPS + PL group, LPS + LL-37 group, and LPS + PL/LL-37 group. BMSCs were seeded in 6-well plates at a density of 5 × 10^4^ cells per well and cultured in high-glucose DMEM complete medium for 24 h at 37 °C with 5 % CO_2_ in a cell culture incubator. Subsequently, the medium was removed and replaced with 2 mL of the prepared osteogenic induction solution (comprising 1 mol/L β-glycerophosphate, 50 mg/mL L-ascorbic acid, and 1 mmol/L dexamethasone), followed by continued incubation in a dark environment. The extracts were refreshed every 3 days. ALP staining was conducted after 7 days of osteogenic induction, while ARS staining was performed after 21 days. Observations were made using a stereomicroscope.

### 2.5 *In vivo* evaluation of PL/LL-37 for periodontal tissue regeneration in SD rats with diabetic periodontitis

#### 2.5.1 The experimental diabetic periodontitis model

The animal protocols for this study were approved in accordance with the guidelines of the Ethical Committee of Anhui Medical University (LLSC20232193). Thirty-five male Sprague-Dawley (SD) rats, aged 5 weeks and weighing 100–150 g, were obtained and housed at the Animal Experiment Center of Anhui Medical University. The animals underwent a one-week acclimatization period prior to the experiment and were randomly assigned into seven groups (n = 5 per group): control group (C), diabetic group (D), periodontitis group (P), untreated diabetic periodontitis group (DP), PL-treated diabetic periodontitis group (DP + PL), LL-37-treated diabetic periodontitis group (DP + LL-37), and PL/LL-37-treated diabetic periodontitis group (DP + PL/LL-37).

Rats were intraperitoneally anesthetized with 0.2 mL/100 g body weight of 3.0 % pentobarbital sodium solution. In the C group and P group, rats were fed a standard diet ad libitum. To induce diabetes, rats were fed a high-sugar and high-fat diet for 8 weeks followed by injection of Streptozotocin (STZ, Sigma) at a dose of 35 mg/kg body weight. Blood glucose levels were measured at 72 h, one week, and two weeks after STZ injection, considering random blood glucose ≥16.7 mmol/L as successful modeling of diabetes induction. To induce periodontitis, the gingiva of the rats was gently separated using a dental probe. Then, a 4-0 ligature was placed through the interproximal space on both sides of the second molar and fixed around the neck region. Finally, *P. gingivalis*-LPS solution supplementation was applied. Ligatures maintenance and *P. gingivalis*-LPS supplementation were performed every 2 days for a total duration of 2 weeks before removal. The PL (10 mg/mL), LL-37 (100 μg/mL) and PL/LL-37 (10 mg/mL PL, 100 μg/mL LL-37) solution were individually mixed with Pluronic® F-127 (Sigma-Aldrich) to form a temperature-sensitive hydrogels for the convenience of injection and to ensure the efficacy of the drug. Pluronic® F127 was a liquid phase at low temperatures (≤10 °C) but converting into a gel at temperatures over 20 °C and formed a stable bio membrane at 37 °C. Subsequently, after ligature removal, different thermosensitive hydrogels containing various drugs were locally injected around both maxillary second molars every 2 days for a duration of 2 weeks.

At the conclusion of the experimental treatments, all animals were euthanized. Maxillae, encompassing all 3 M, alveolar bone, and attached gingival tissue, were harvested and fixed in a 4 % paraformaldehyde solution for subsequent Micro-computed Tomography (Micro-CT) analyses, histological examination, and Immunohistochemical (IHC) staining.

#### 2.5.2 Micro-CT analysis

The bilateral maxillary samples of rats were scanned at a resolution of 9 μm using Micro-CT (SkyScan 1176, Bruker, Germany). Following completion of the scanning process, a manual selection was made for the interval of interest and CT analysis software was employed to perform a 3D reconstruction in order to observe the alveolar bone.

The vertical distance between the cementoenamel junction (CEJ) and the alveolar bone crest (ABC) on the buccal proximal-medial, central, and distal aspects of the maxillary second molar was measured using ImageJ software to assess the extent of periodontal bone destruction.

Bilateral mesial and distal alveolar bone volume fractions (BV/TV), bone mineral density (BMD), trabecular bone number (Tb.N), trabecular bone thickness (Tb.Th), and trabecular bone separation (Tb.Sp) were analyzed in a region comprising 20 layers both anteriorly and posteriorly within the maximal cross-section of the root canals of the maxillary second molar.

#### 2.5.3 Histological analysis

The harvested maxillae were initially fixed in a 4 % paraformaldehyde solution for 48 h and subsequently decalcified in a 10 % EDTA solution at 4 °C for a duration of 8 weeks. Following decalcification, the specimens underwent dehydration, paraffin embedding, and sectioning at a thickness of 4 μm. The sections were then mounted on glass slides and subjected to Hematoxylin and eosin staining (HE, Servicebio) to evaluate inflammatory infiltration in the periodontal tissue of the maxillary second molar. Tartrate-resistant acid phosphatase staining (TRAP, Servicebio) was employed to quantify osteoclasts, while Masson staining (Servicebio) was utilized to assess collagen fibers.

#### 2.5.4 IHC

The rat maxillary second molars were subjected to IHC analysis to detect markers associated with anti-inflammatory, osteogenic, apoptotic, and diabetes-protective processes. This investigation aimed to evaluate the potential of PL/LL-37 in mitigating periodontal tissue destruction, promoting periodontal tissue regeneration, and elucidating underlying mechanisms. The examined immunomarkers included TNF-α (1:100, Abcam), IL-1β (1:100, Abcam), IL-6 (1:100, Abcam), and P-p65 (1:100, HuaBio) for anti-inflammatory responses; runt-related transcription factor 2 (RUNX2) as well as bone sialoprotein (BSP) and osteoprotegerin (OPG) [RUNX2 (1:100, HuaBio); BSP (1:100, Affinity); OPG (1:100, Affinity)] for osteogenesis; B-cell lymphoma 2 (Bcl-2) and Bcl-2-associated X protein (Bax) (both at a dilution of 1:100 from Affinity) for apoptosis; RAGE (1:100, Abcam) for diabetes protection.

### 2.6 Statistical analysis

The statistical analysis for this study was performed using Prism version 9.0 (GraphPad Software, USA). The data underwent one-way analysis of variance (ANOVA), and the results were presented as mean ± standard deviation. Significance levels were indicated as follows: ∗ for P < 0.05, ∗∗ for P < 0.01, ∗∗∗ for P < 0.001, and ∗∗∗∗ for P < 0.0001. The experiments were replicated three times to ensure result reliability.

## 3 Results and discussions

### 3.1 The antimicrobial properties and cytocompatibility of LL-37 improved by PL

The total phenol content in PL was quantified using phloroglucinol as a standard. The standard curve established a regression equation of C = 213.66A-31.665 (R^2^ = 0.9939), where C was the total phenol concentration, and A was the absorbance. The total phenol content in the PL used was found to be 15.84582 μg phloroglucinol equivalent/mg. Future studies may benefit from using polyphenol-rich PL to unlock its full potential. Conventional methods for extracting PL typically involve the utilization of solvents, such as ethanol, acetone, methanol, or their aqueous mixtures. However, these approaches encounter challenges pertaining to environmental impact, sustainability, solvent storage and disposal. To address these limitations, alternative technologies including supercritical fluids, ultrasound-assisted microwave extraction and deep eutectic solvents are being investigated to enhance extraction efficiency and sustainability while minimizing environmental consequences [46].

**Fig. 1:**
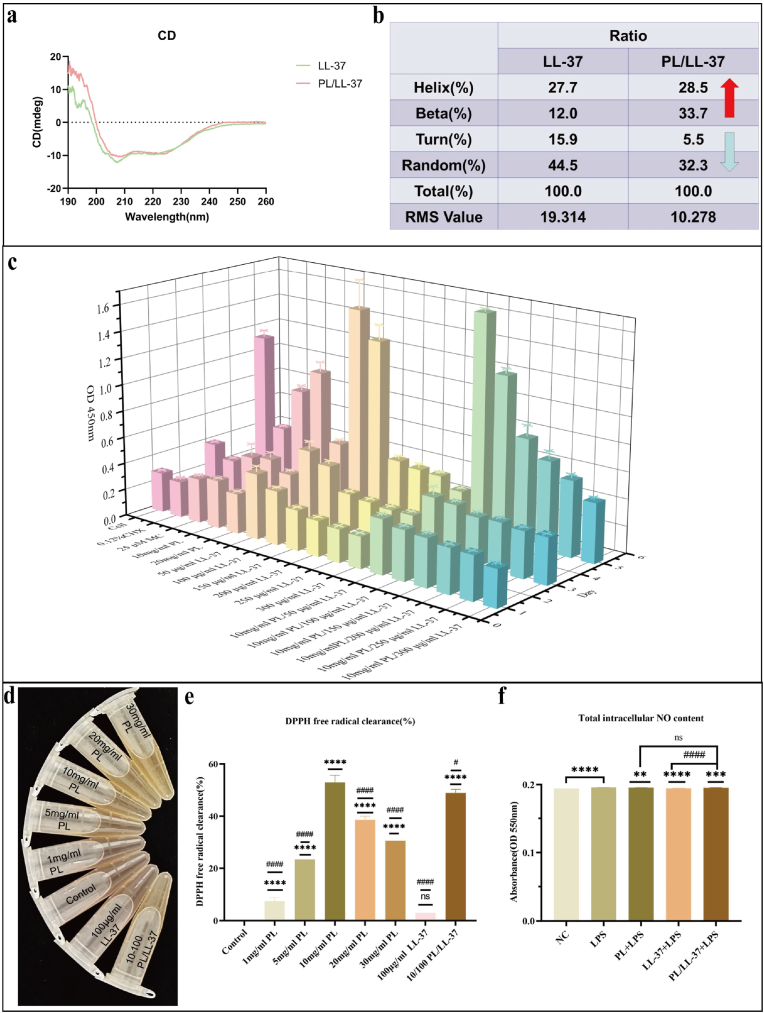
The secondary structure analysis, biosafety and antioxidant properties evaluation of PL/LL-37. (a) The CD results of LL-37 and PL/LL-37. (b) The proportion of protein secondary structure of LL-37 and PL/LL-37, indicating changes in the secondary structure when PL was added to LL-37. (c) CCK-8 assay in MC3T3-E1 under different concentrations of PL, LL-37 and PL/LL-37 over time. (d) Color change of DPPH solution, illustrating the antioxidant properties of PL, LL-37, and PL/LL-37. Asterisks (∗) indicate comparisons with Control group, while hashtags (#) denote comparisons with 10 mg/mL PL group. (e) DPPH free radical clearance rate of PL, LL37 and PL/LL-37. (f) Total intracellular NO content of PL, LL-37, and PL/LL-37. Asterisks (∗) indicate comparisons with NC group, while hashtags (#) denote comparisons with PL/LL-37 group. (For interpretation of the references to color in this figure legend, the reader is referred to the Web version of this article.)

The sequence purity and authenticity of LL-37 were determined by HPLC (Fig. S1a) and its molecular mass was determined by MS (Fig. S1b), respectively. We synthesized the antimicrobial peptide LL-37 with a purity of 97.55 % and a molecular weight of 4493.37. The CD chromatography results of LL-37 and PL/LL-37 were depicted in Fig. 1a. Subsequent protein secondary structure analysis was conducted based on the CD results, and the outcome was presented in Fig. 1b. Compared to LL-37, PL/LL-37 exhibited an increased proportion of α-helix and β-fold structures, accompanied by a decreased proportion of β-turn and random coil. Peptides with an α-helix structure are amphiphilic, featuring one end as a hydrophilic region and the corresponding end as a hydrophobic region [50]. The hydrophilic-lipophilic structure serves as the foundation for the antimicrobial function of antimicrobial peptides. Previous studies have indicated that α-helix conformation facilitates a stronger binding between antimicrobial peptides and cell membranes, establishing a significant correlation between helicity and antibacterial activity [51]. Peptides adopting a β-folded structure possess disulfide bonds that contribute to stabilizing the peptide structure, thereby preserving its integrity during cell membrane penetration and enhancing bactericidal functionality. Consequently, modifying the proportion of this secondary structure in PL/LL-37 leads to increased stability of the overall structure, ultimately improving its antibacterial properties.

As a type of seaweed extract, PL possesses green and safe characteristics. A study demonstrated that Ecklonia cava phlorotannins, as a novel food source, could be safely incorporated into food supplements with a maximum intake of 163 mg per day for adolescents aged 12–14 years, 230 mg for adolescents over 14 years, and 263 mg for adults [52]. Previous investigations have indicated the cytotoxicity of LL-37 [[53], [54]]. Therefore, MC3T3-E1, BMSCs and RAW 264.7 cells were used to assess the cytocompatibility of PL, LL-37 and PL/LL-37. The results of CCK-8 in MC3T3-E1 revealed that 20 mg/mL PL, CHX and MC cytotoxicity, especially in 5 d (Fig. 1c). However, the addition of 10 mg/mL PL mitigated the cytotoxicity of LL-37, especially at high concentrations (150–300 μg/ml) of LL-37. This suggests that PL may interact with LL-37 and reduce its free concentration in the medium. Overall, PL/LL-37 (10 mg/mL PL, 50 μg/mL and 100 μg/mL LL-37) exhibited superior biocompatibility in 1, 3, and 5 days. Furthermore, the results of CCK-8 assays in BMSCs (Fig. S2a) and RAW 264.7 cells (Fig. S2b) were consistent with those observed in MC3T3-E1 cells. Additionally, a study demonstrated that the minimum inhibitory concentration (MIC) of LL-37 against oral specialized anaerobic suspension and parthenogenetic anaerobic suspension was determined to be 100 μg/mL [55]. Therefore, we proposed to use 100 μg/mL as the experimental concentration of LL-37, which would continue to be verified in the subsequent antimicrobial evaluation.

### 3.2 Anti-oxidation properties of PL/LL-37

In the DPPH assay, DPPH exhibited a purple color in an alcohol solution, which changed to light yellow upon addition of PL (Fig. 1d). Fig. 1d demonstrated that 1 mg/mL PL, 100 μg/mL LL-37, and the control group exhibited a light purple color, while 5 mg/mL PL, 10 mg/mL PL, 20 mg/mL PL, 30 mg/mL PL, and PL/LL-37 (10 mg/mL PL, 100 μg/mL LL-37) appeared as light yellow. These results indicated that 5–30 mg/mL PL and PL/LL-37 (10 mg/mL PL, 100 μg/mL LL-37) exhibited visible antioxidant effects. The specific clearance rates of DPPH free radical for each group were presented in Fig. 1e. Among them, the highest DPPH free radical clearance rate was observed in 10 mg/mL PL (52.89 %), followed by PL/LL-37 (10 mg/mL PL, 100 μg/mL LL-37, 48.90 %). Importantly, it should be noted that there was no linear positive correlation between the scavenging rate of DPPH free radicals and increasing concentration of PL. The rates for 20 mg/mL PL (38.52 %) and 30 mg/mL PL (30.54 %) were lower than that of 10 mg/mL PL, and precipitation was observed in the groups with 20 mg/mL and 30 mg/mL PL concentrations. This observation suggests that the high concentration of PL may not be fully dissolved, thereby affecting its antioxidant properties. Considering the results obtained from evaluating biocompatibility and antioxidant properties of PL, a final experimental concentration of 10 mg/mL was selected. Furthermore, measurements of intracellular NO content (Fig. 1f) indicated that the use of 10 mg/mL PL, along with LL-37 at a concentration of 100 μg/mL or in combination as PL/LL-37 (10 mg/mL PL, 100 μg/mL LL-37), effectively reduced intracellular NO content during *P. gingivalis*-LPS stimulation, demonstrating statistically significant differences. The present study demonstrated that PL effectively suppressed the production of LPS-induced NO and prostaglandin E2 (PGE2) through a potential mechanism involving downregulation of inducible nitric oxide synthase (iNOS) and cycloheximide 2 proteins, thereby exerting antioxidant and anti-inflammatory effects [56]. These findings are consistent with our experimental results.

#### 3.2.1 Disc diffusion

The qualitative experimental results depicting the sensitivity of PL, LL-37, and PL/LL-37 towards *P. gingivalis* were presented in Fig. S3a. The area highlighted within the green circle in Fig. S3a represented the region of the inhibitory ring, which illustrates the drug's inhibitory effect on *P. gingivalis.* A larger ring signified a more pronounced inhibitory effect, assuming all other factors remain constant. Upon adding 20 μL of LL-37 and PL/LL-37 to the blood agar plate for bacterial stimulation over a period of 7 days, it was observed that both LL-37 (Fig. S3a2) and PL/LL-37 (Fig. S3a5,6,8–10) exhibited significant bacteriostatic ability by forming an inhibitory ring with a diameter of 11 mm on the blood agar plate. Interestingly, as the concentration of LL-37 increased within PL/LL-37, there was minimal alteration in the diameter of the inhibition ring, suggesting that this inhibitory effect might not be directly correlated to the concentration of LL-37 within PL/LL-37. Previous studies had reported an MIC value of 100 μg/mL for LL-37 against obligatory and facultative oral anaerobe suspensions [55]. Another study had demonstrated that the MIC of LL-37 against *P. gingivalis* was 20 μM (≈90 μg/mL) [57]. Fig. S3a5 illustrated the potent antibacterial activity of PL/LL-37 (10 mg/mL PL, 50 μg/mL LL-37), suggesting that the inclusion of PL might enhance the antimicrobial efficacy of LL-37 against *P. gingivalis*. To investigate the correlation between different concentrations of PL/LL-37 and the antibacterial properties against *P. gingivalis*, additional quantitative assessments were conducted on diluted coated plates.

#### 3.2.2 The ability of PL/LL-37 to resist planktonic bacteria

**Fig. 2:**
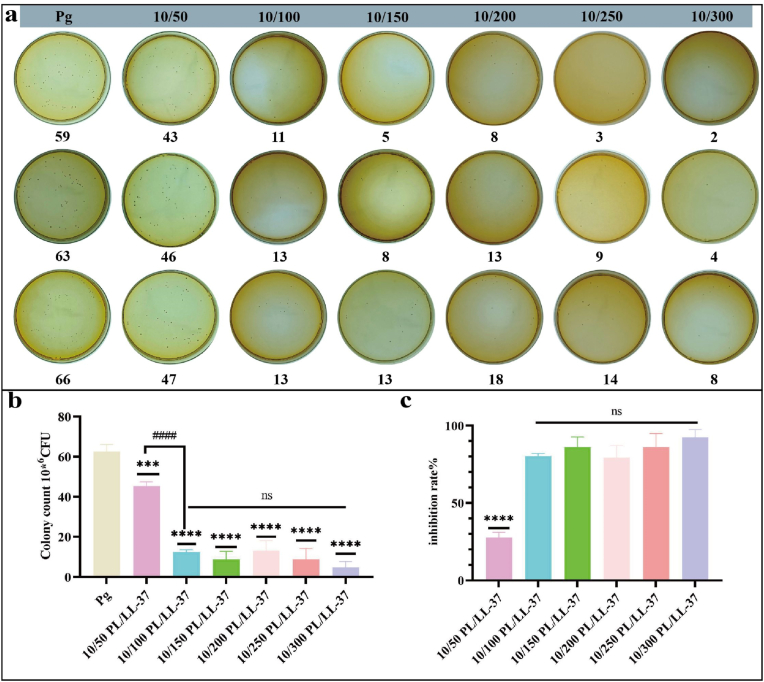
Antimicrobial effect of different concentrations of PL/LL-37. (a) Diluted coated plate counts. (b) Statistical analysis of colony counts. (c) Statistical analysis of inhibition rate. Asterisks (∗) indicate comparisons with Pg group, while hashtags (#) denote comparisons with 10/100 PL/LL-37 group.

The results of diluted coated plate, which were counted for different concentrations of PL/LL-37 co-cultured with *P. gingivalis*, were presented in Fig. 2a. It could be observed from Fig. 2a that the antibacterial efficacy of PL/LL-37 (10 mg/mL PL, 100 μg/mL LL-37) was superior to that of PL/LL-37 (10 mg/mL PL, 50 μg/mL LL-37). However, there was no significant difference in the antibacterial performance between PL/LL-37 with higher concentrations of LL-37 and PL/LL-37 (10 mg/mL PL, 100 μg/mL LL-37). Statistical analysis also confirms the absence of any significant differences as shown in Fig. 2b. The inhibition rate was calculated and depicted in Fig. 2c. As evident from Fig. 2b and c, the antibacterial activity exhibited by PL/LL-37 (10 mg/mL PL, 100 μg/mL LL-37) surpassed that demonstrated by PL/LL-37 (10 mg/mL PL, 50 μg/mL LL-37; P < 0.0001), although no statistical differences were observed among various concentrations of PL/LL-37 (10 mg/mL PL, 15–300 μg/mL LL-37). Therefore, we selected a concentration of PL/LL-37 (10 mg/mL PL, 100 μg/mL LL-37) for subsequent experimental studies.

**Fig. 3:**
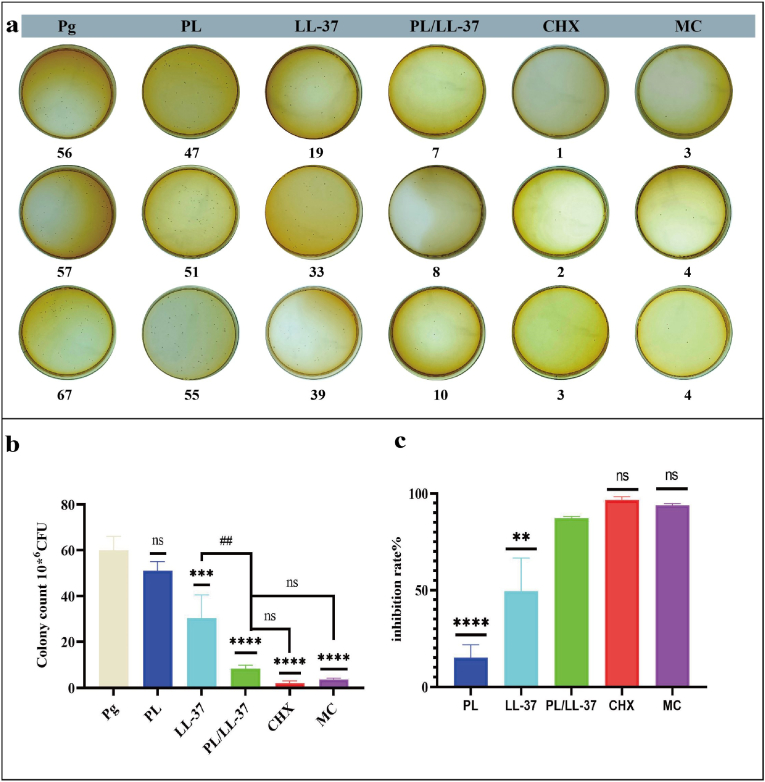
Antimicrobial effect of PL, LL-37 and PL/LL-37. (a) Diluted coated plate counts. (b) Statistical analysis of colony counts. Asterisks (∗) indicate comparisons with Pg group, while hashtags (#) denote comparisons with PL/LL-37 group. (c) Statistical analysis of inhibition rate. Statistical analyses were conducted to compare each group with the PL/LL-37 group.

The results presented in Fig. 3a demonstrated the bactericidal properties of PL/LL-37 when co-cultured with *P. gingivalis*, surpassing the individual efficacy of LL-37 alone but falling short compared to CHX and MC. Statistical analysis (Fig. 3b) and the inhibition rate (Fig. 3c) further confirmed that PL/LL-37 exhibited superior antibacterial performance compared to LL-37 alone, with a significant difference observed between the two (P < 0.01). Notably, there was no statistical distinction between PL/LL-37, CHX, and MC, indicating the commendable antibacterial potential of PL/LL-37.

#### 3.2.3 Inhibition of bacterial growth

The growth curve of *P. gingivalis* was depicted in Fig. S3b. Except for the 4th day, the antibacterial activity of PL/LL-37 exhibited superior efficacy compared to LL-37 alone, thereby providing further evidence that the inclusion of PL enhanced the antibacterial performance of LL-37.

#### 3.2.4 Inhibition of bacterial adhesion and biofilm formation

**Fig. 4:**
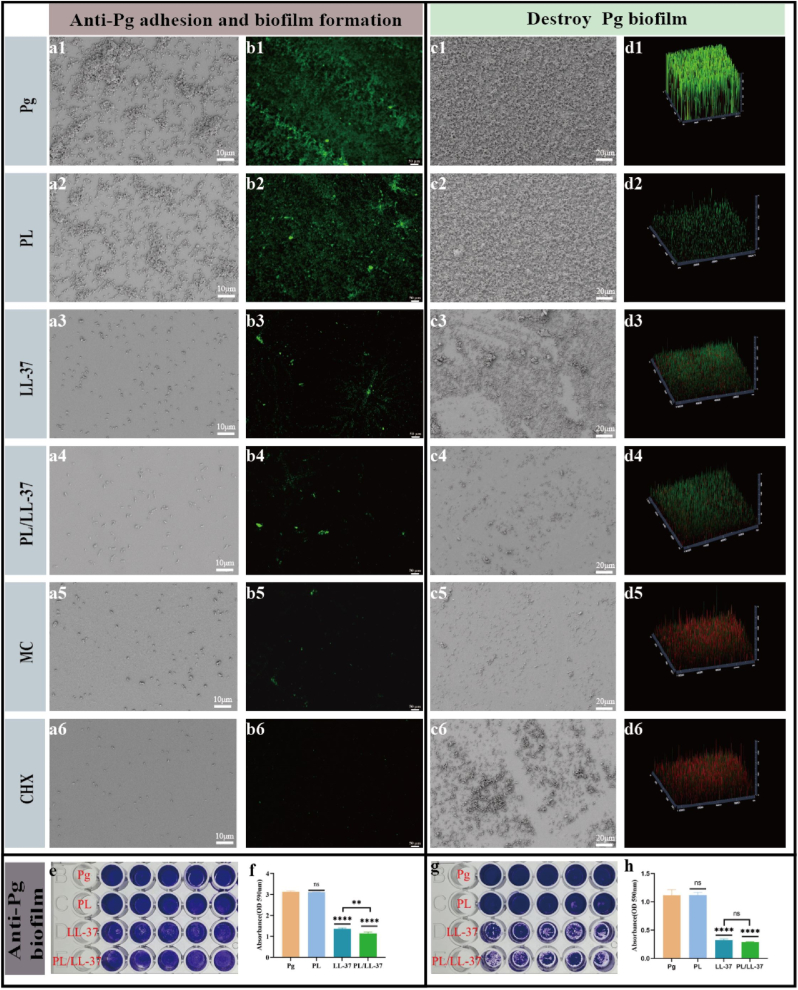
Inhibition of *P. gingivalis* adhesion, biofilm formation, disruption of formed *P. gingivalis* biofilms and antimicrobial mechanism. (a1-a6) SEM images of *P. gingivalis* adhesion conditions. (b1-b6) CLSM images of *P. gingivalis* adhesion conditions. (c1-c6) SEM images of *P. gingivalis* biofilms. (d1-d6) CLSM images of *P. gingivalis* biofilms. (a1, b1, c1, d1) control. (a2, b2, c2, d2) 10 mg/ml PL. (a3, b3, c3, d3) 100 μg/mL LL-37. (a4, b4, c4, d4) PL/LL-37 (PL: 10 mg/mL, LL-37: 100 μg/mL). (a5, b5, c5, d5) 25 μM MC. (a6, b6, c6, d6) 0.12 % CHX. (e) Crystal violet staining photographs for observation of inhibiting *P. gingivalis* biofilms formation. (f) Statistical analysis of OD value of biofilm dissolution after crystal violet staining. (g) Crystal violet staining photographs for observation of destroying the formed *P. gingivalis* biofilms. (h) Statistical analysis of OD value of biofilm dissolution after crystal violet staining. (For interpretation of the references to color in this figure legend, the reader is referred to the Web version of this article.)

The inhibitory effects on bacterial adhesion were illustrated in Fig. 4 a1-b6. PL (Fig. 4a2, b2) exhibited a modest inhibitory effect on *P. gingivalis* adhesion, while both LL-37 (Fig. 4a3, b3) and PL/LL-37 (Fig. 4a4, b4) demonstrated the ability to inhibit *P. gingivalis* adhesion; however, their efficacy was slightly inferior to MC (Fig. 4a5, b5) and CHX (Fig. 4a6, b6). Notably, the inhibition of *P. gingivalis* adhesion by PL/LL-37 surpassed that of LL-37 alone. The results pertaining to the inhibition of *P. gingivalis* biofilm formation were presented in Fig. 4e and f. Both LL-37 (P < 0.0001) and PL/LL-37 (P < 0.0001) effectively inhibited bacterial biofilm formation. Furthermore, PL/LL-37 demonstrates superior efficacy in inhibiting the formation of *P. gingivalis* biofilms compared to LL-37 alone (P < 0.01).

#### 3.2.5 Anti-biofilm ability

The efficacy of PL/LL-37 in eradicating *P. gingivalis* biofilm was assessed using SEM, Live/Dead staining, and crystal violet staining, and the results were shown in Fig. 4c, d, g, h. It was evident that PL/LL-37 exhibited a significant capability to disrupt *P. gingivalis* biofilms. The bacterial biofilms were unbroken in the *P. gingivalis* and PL groups, while the bacterial biofilms are broken in the LL-37 group and PL/LL-37 group. Crystal violet staining results demonstrated that both LL-37 (P < 0.0001) and PL/LL-37 (P < 0.0001) exerted considerable effects on the eradication of established bacterial biofilms without any statistically significant difference between them. Meanwhile, the results of the BCA evaluation for PL/LL-37 regarding its efficacy in disrupting P. *gingivalis* bacterial biofilm was presented in Fig. S3c. The protein content in the supernatant from both the LL-37 and PL/LL-37 groups showed a significant increase compared to the P. *gingivalis* group, with statistical differences observed (P < 0.05). This indicated that both LL-37 and PL/LL-37 were capable of disrupting P. *gingivalis* bacterial biofilm and facilitating the release of cellular contents.

#### 3.2.6 Antibacterial mechanisms of PL/LL-37

**Fig. 5:**
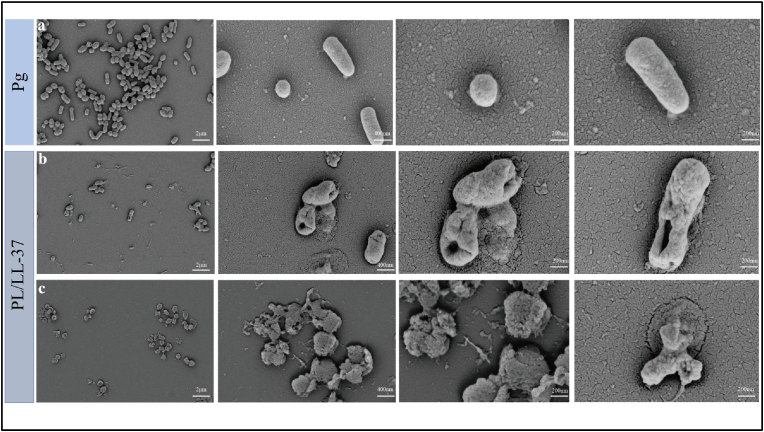
The Pg bacterial morphology observed under SEM in both the Pg group and the PL/LL-37 group. (a) The normal morphology of *P. gingivalis* bacteria exhibited characteristics similar to micrococcus and brevibacterium, with a smooth and glossy surface accompanied by distinct secondary division. (b) Upon treatment with PL/LL-37, significant damage to the *P. gingivalis* bacterial cell membrane was observed, resulting in the formation of lacunae of the bacterial plasma membrane. (c) Upon treatment with PL/LL-37, significant damage to the *P. gingivalis* bacterial cell membrane was observed, resulting in the formation of rupture of the bacterial plasma membrane, leading to leakage of intracellular contents.

The action mode of PL/LL-37 on *P. gingivalis* bacteria was directly observed using SEM to investigate its impact on the external morphology of the bacteria (Fig. 5). The normal morphology of *P. gingivalis* bacteria (Fig. 5a) exhibited characteristics similar to micrococcus and brevibacterium, with a smooth and glossy surface accompanied by distinct secondary division. Upon treatment with PL/LL-37, significant damage to the *P. gingivalis* bacterial cell membrane was observed, resulting in the formation of lacunae (Fig. 5b) and rupture of the bacterial plasma membrane, leading to leakage of intracellular contents (Fig. 5c). Previous studies had demonstrated that LL-37 effectively inhibited and disrupted bacterial biofilms through ion channel formation while also exhibiting rapid binding affinity towards LPS present on the bacterial membrane surface, thereby neutralizing bacteria and displaying potent antibacterial properties [58]. Our findings demonstrated a resemblance to the antimicrobial mechanism of LL-37, implying that PL/LL-37 might also exhibit rapid bactericidal activity through the formation of ion channels on microbial cell membranes and binding to bacterial membrane endotoxins.

### 3.3 PL/LL-37 promoted the conversion of *P. Gingivalis*-LPS-stimulated RAW264.7 from M1 to M2 type and reduced the accumulation of RAGE

#### 3.3.1 qRT PCR

**Fig. 6:**
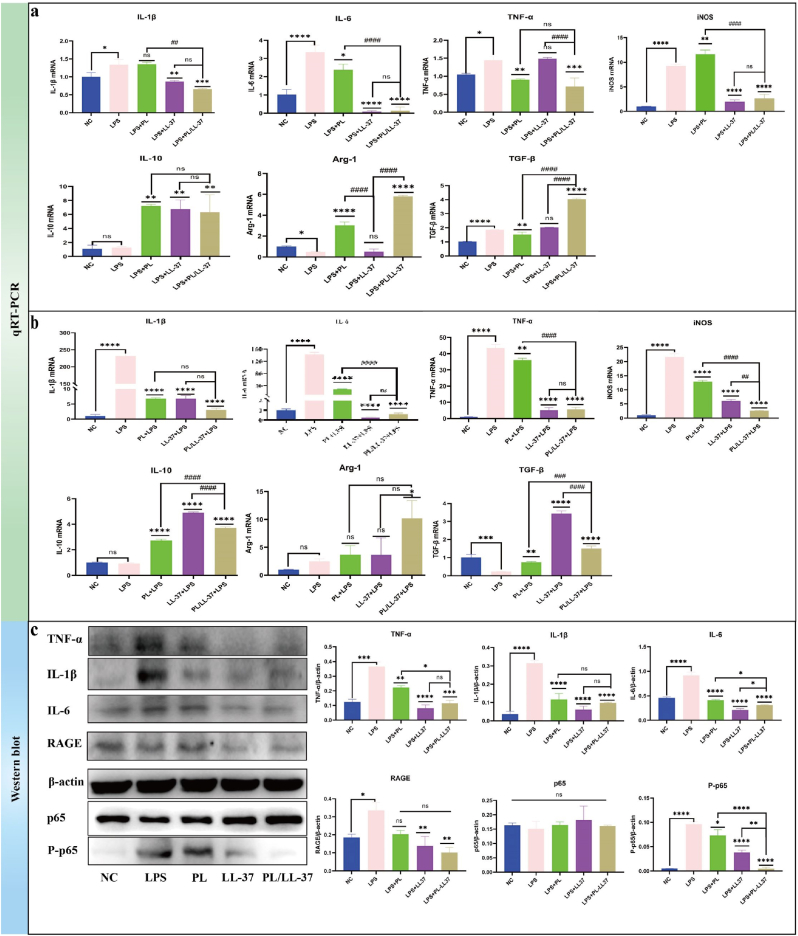
Evaluation of immunomodulatory and diabetes protective properties. (a) qRT PCR results of RAW264.7 cells stimulated by *P. gingivalis*-LPS for 4 h and then treated with PL/LL-37 for 24 h. (b) qRT PCR results of RAW264.7 cells treated with PL/LL-37 for 24 h and then stimulated with *P. gingivalis*-LPS for 4 h. (c) The results of WB.

The impact of PL/LL-37 on the cytokine release by M1 and M2 macrophages was further investigated using qRT-PCR. Inflammatory cytokines produced by M1 macrophages included IL-1β, IL-6, TNF-α, and iNOS, while anti-inflammatory factors released by M2 macrophages consisted of Arginase-1 (Arg-1), Transforming Growth Factor-beta (TGF-β), and IL-10. Fig. 6a illustrates the qRT PCR results obtained from RAW264.7 cells stimulated with *P. gingivalis*-LPS and subsequently treated with PL/LL-37. The expression levels of inflammatory factors were significantly higher in the LPS group compared to the control group, confirming successful inflammation modeling. Notably, PL/LL-37 exhibited significant inhibition on four inflammatory factors (IL-1β: P < 0.001; IL-6: P < 0.0001; TNF-a: P < 0.001; iNOS: P < 0.0001). Importantly, PL/LL -37 demonstrated superior inhibitory effects on IL - 1 β compared to PL (P < 0 00.01), as well as better suppression of TNF - α than LL -37 (P < 0.0001). The production of anti-inflammatory cytokines, particularly Arg-1 (P < 0.0001) and IL-10 (P < 0.01), could be significantly increased by PL treatment. Moreover, the combined use of PL/LL-37 demonstrated superior efficacy in enhancing the expression of Arg-1 (P < 0.0001) and TGF-β (P < 0.0001) compared to individual treatments with PL or LL-37 alone. However, no significant difference was observed in promoting the expression of IL-10 between these treatments. Overall, PL/LL-37 exhibited a potent anti-inflammatory effect, with PL primarily improving the production of anti-inflammatory factors while LL-37 mainly reducing pro-inflammatory factor production. The results presented in Fig. 6b demonstrate that RAW264.7 cells were treated with PL/LL-37 for 24 h, followed by stimulation with *P. gingivalis*-LPS for 4 h. Consistently, the expression levels of IL-1β, IL-6, TNF-α and iNOS, four inflammatory cytokines, were significantly higher in the LPS group compared to the control group (p < 0.05), confirming successful construction of the inflammation model. Notably, both PL and LL-37 individually as well as their combination (PL/LL-37) exhibited potent inhibitory effects on inflammatory factor production (p < 0.0001). Moreover, PL/LL-37 also demonstrated a certain degree of efficacy in promoting the expression of anti-inflammatory factors such as TGF-β and IL-10 (p < 0.0001).

#### 3.3.2 WB

The results of WB (Fig. 6c) were consistent with those obtained from qRT-PCR. PL/LL-37 demonstrated a significant ability to attenuate the protein expression levels of key inflammatory mediators, including IL-1β, IL-6, and TNF-α, while also down-regulating the expression of RAGE and P-p6. Hence, we postulated that the anti-inflammatory effects exerted by PL/LL-37 were mediated through the down-regulation of RAGE and P-p65 signaling pathways. Furthermore, our findings indicated that PL/LL-37 exhibited superior efficacy in reducing RAGE and P-p65 levels compared to either PL or LL-37 alone, particularly in terms of suppressing P-p65 expression. Consequently, it could be concluded that PL/LL-37 possesses enhanced anti-inflammatory properties when compared to individual components such as PL or LL-37.

Diabetic patients experience chronic hyperglycemia, which leads to the exacerbation and dysregulation of the inflammatory response and subsequent destruction of periodontal tissue through various mechanisms. These include up-regulation of inflammatory factor production, activation of AGE-RAGE signaling pathway [59], modulation of apoptosis, and regulation of Polymorphonuclear Neutrophils (PMN) function. Notably, periodontal inflammation can induce insulin resistance. The key factors linking diabetes with periodontitis encompass AGEs, IL-1β, IL-6, and TNF-α [59]. In terms of anti-inflammatory effects, our study primarily focused on IL-1β, IL-6, TNF-α cytokines, as well as RAGE and the NF-κB P-p65 pathway. Notably, a study demonstrated that PL effectively suppressed the production of NO and PGE2 induced by endotoxin through down-regulating the expression of iNOS synthase and cyclocycide 2 proteins, thereby exhibiting potent antioxidant and anti-inflammatory activities [56]. Additionally, PL was found to attenuate AGEs production [60] and exerted anti-inflammatory effects via modulation of the NF-κB pathway [61]. These findings are consistent with our present study.

The periodontal tissue expresses the antimicrobial peptide LL-37, and alterations in LL-37 levels have been associated with periodontal disease. A study demonstrated that high doses of LL-37 inhibited DNA synthesis, attenuated LPS-induced cytokine and chemokine production, and reduced cell proliferation in human periodontal ligament cells by promoting apoptosis [62]. Another study revealed a positive correlation between salivary LL-37 and salivary AGE as well as Hemoglobin A1C (HbA1c) [63]. The level of LL-37 in the periodontal tissue of patients with type 2 diabetes mellitus was found to be increased [63]. Despite elevated levels of the antimicrobial peptide LL-37, individuals with type 2 diabetes mellitus remain susceptible to periodontal infections, possibly due to the cytotoxic effects exerted by high concentrations of LL-37 on host cells [63]. Although high concentrations of LL-37 have anti-inflammatory effects, they may exacerbate periodontal tissue destruction due to their toxicity to host cells. In this study, the addition of PL reduced the cytotoxicity of high concentration LL-37. When applied in vitro, PL/LL-37 may have a better effect on treating diabetic periodontitis. Overall, our findings suggest that PL/LL-37 has multifaceted impacts on periodontal tissue health, including down-regulating pro-inflammatory pathways, antioxidant effects, reducing RAGE accumulation and promoting anti-inflammatory and tissue repair factors. These findings align with potential therapeutic benefits for periodontal conditions.

#### 3.3.3 ALP and ARS staining

**Fig. 7:**
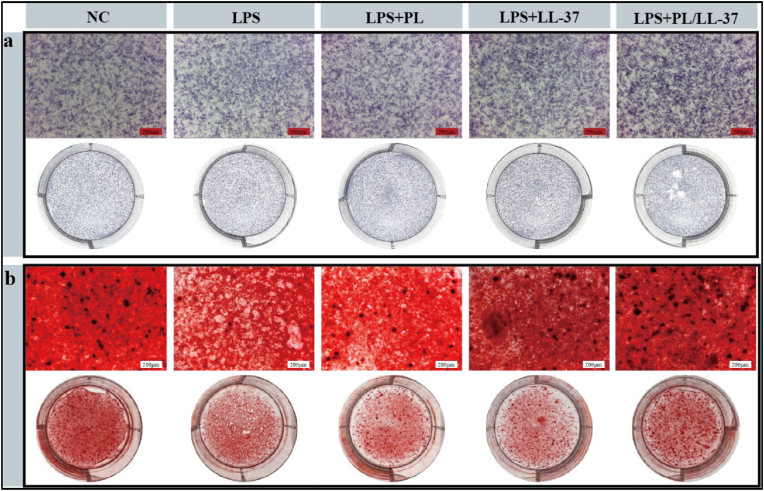
Osteogenic differentiation of BMSCs induced by PL/LL-37. (a) ALP staining on 7 days of osteogenic induction of BMSCs with hydrogel osteogenesis extracts. (b) ARS staining on 21 days of osteogenic induction of BMSCs with hydrogel osteogenesis extracts.

ALP staining (Fig. 7a) showed that the LL-37 and PL/LL-37 groups stained more deeply compared to the control and LPS groups and the PL group. The PL/LL-37 group stained darker compared to the LL-37 group. There was a clear trend observed in the results of ARS staining (Fig. 7b), indicating that both the LPS + PL group, LPS + LL-37 group, and LPS + PL/LL-37 group exhibited significantly deeper staining compared to the LPS group. Among these groups, the LPS + PL/LL-37 group demonstrated the deepest staining. In summary, PL/LL-37 effectively promoted the osteogenic differentiation of BMSCs, resulting in a significant increase in calcium nodule formation and enhanced production of ALP. Notably, this bone-enhancing effect was superior to that observed with either PL or LL-37 alone.

### 3.4 PL/LL-37 promotes periodontal tissue regeneration in rats with diabetic periodontitis

#### 3.4.1 Successful modelling of diabetic periodontitis in SD rats

The diabetic periodontitis model was successfully established in SD rats, as demonstrated in Fig. S4. Compared to non-diabetic SD rats, diabetic SD rats exhibited a yellowish and rough hair color along with reduced body weight (Figs. S4a and S4b). Additionally, the random blood glucose levels of diabetic SD rats were ≥16.7 mmol/L, confirming the successful induction of diabetes (Figs. S4c and S4d). Subsequently, rat periodontitis modeling was performed as depicted in Fig. S4e. The presence of alveolar bone resorption around the maxillary second molar indicated successful establishment of periodontitis (Fig. S4f).

#### 3.4.2 Micro-CT analysis of the periodontal bone resorption

**Fig. 8:**
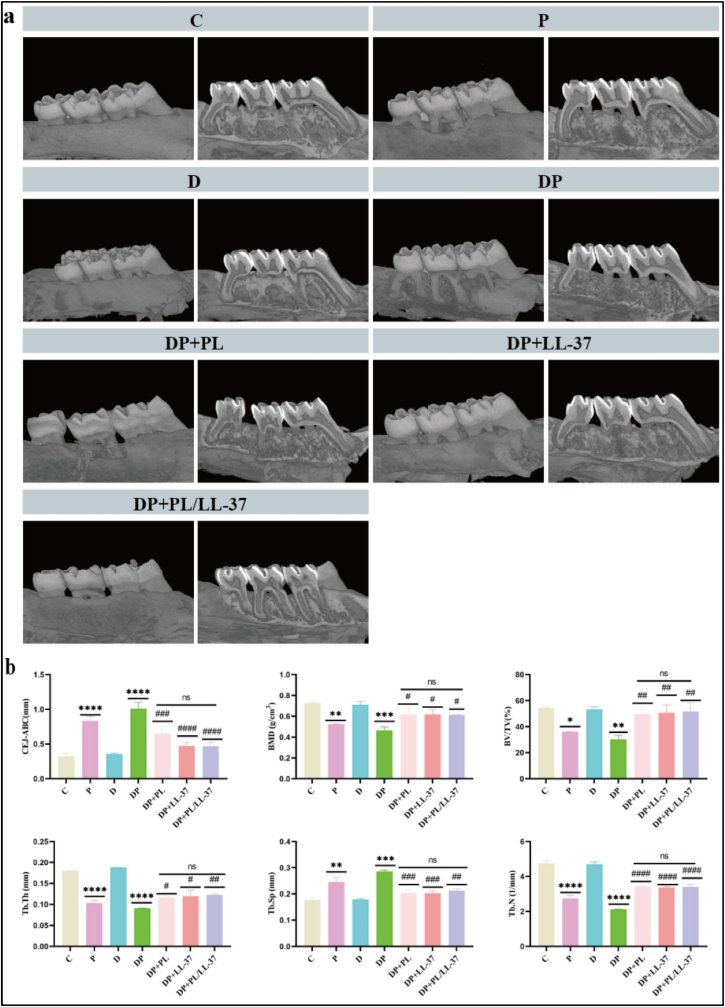
PL/LL-37 reduces periodontal tissue destruction in SD rats with diabetic periodontitis. (a) Determination of the alveolar bone resorption using Micro-CT. (b) Results from the analysis of micro-CT data. The values for CEJ-ABC, BMD, BV/TV, Tb. Th, Tb.Sp, and Tb. N of the alveolar bone in each group are presented along with intergroup comparison results. Asterisks (∗) indicate comparisons with group C, while hashtags (#) denote comparisons with group DP.

The SD rats were euthanized following the successful modeling of diabetic periodontitis for a duration of 2 weeks, and Micro-CT analysis was performed to evaluate the defect in maxillary second molar's periodontal bone tissue (Fig. 8a). Both the P group and DP group exhibited evident bone resorption and exposure of root bifurcation in the maxillary second molar. Treatment with PL, LL-37, or PL/LL-37 resulted in varying degrees of improvement in bone resorption, with the PL/LL-37 group demonstrating the least amount of bone resorption and superior alveolar bone health.

Analysis of Micro-CT measurements (Fig. 8b) revealed a significant increase in the mean CEJ-ABC distance in both the P and DP groups compared to the C group (p < 0.0001), thereby confirming the successful establishment of periodontitis modeling. Notably, the mean CEJ-ABC distance was significantly lower in the DP + PL (p < 0.001), DP + LL-37 (p < 0.0001), and DP + PL/LL-37 groups (p < 0.0001) when compared to the DP group, indicating that PL, LL-37, and PL/LL-37 facilitated periodontal tissue regeneration in rats with diabetic periodontitis. Furthermore, parameters such as BMD, BV/TV, Tb.Th., and Tb.N were significantly increased (p < 0.05; p < 0.01; p < 0.01; p < 0.01; p < 0.0001), while Tb.Sp was significantly decreased (p < 0.01) in the PL/LL-37 group relative to the DP group, suggesting that treatment with PL/LL-37 effectively restored alveolar bone density and volume.

#### 3.4.3 Histological analysis

**Fig. 9:**
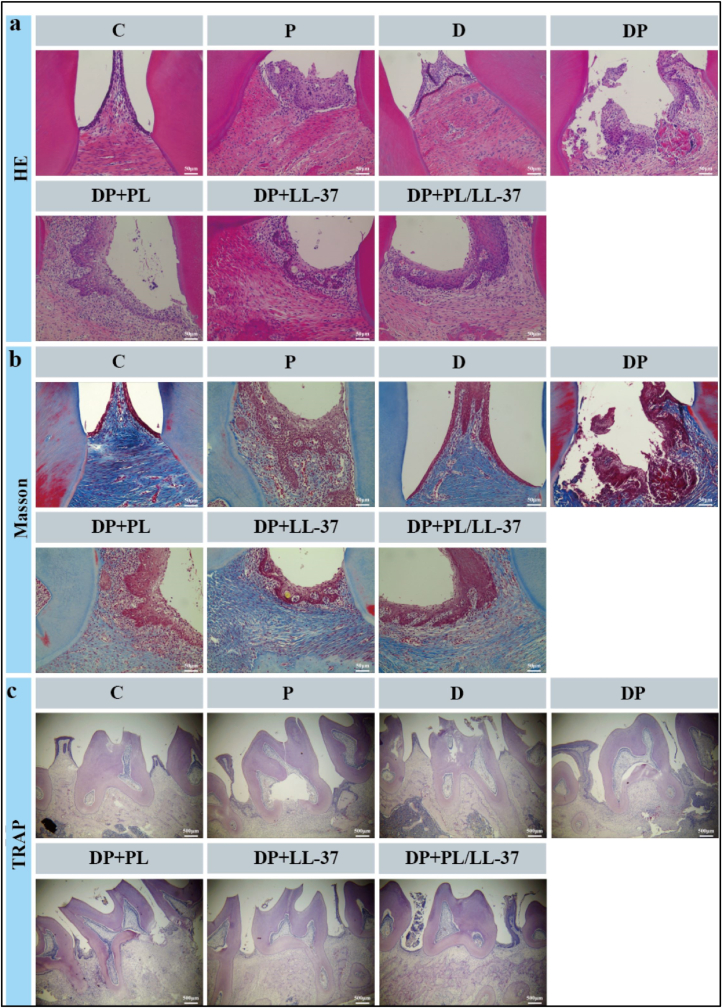
Histologic changes and cytokine expression in the periodontium of the maxillary second molar of periodontitis rats after treatment with different groups of hydrogels. (a) HE staining images of the periodontium after the treatment in decalcified maxilla sections. (b) Masson staining images of the periodontium after the treatment in decalcified maxilla sections. (c) Representative images of osteoclasts staining in the alveolar bone around second molar by TRAP.

The HE staining technique was employed to further assess the inflammatory response and periodontal tissue status in each group (Fig. 9a). In both the P and DP groups, there was a significant increase in the number of gingival inflammatory cells, indicating a persistent state of inflammation and substantial loss of attachment. Moreover, the inflammatory response was more severe in the DP group compared to the P group. Treatment with PL, LL-37, or their combination (PL/LL-37) resulted in a reduction in infiltrating inflammatory cells, mitigated the inflammatory response, and improved periodontitis gum morphology in rats with periodontitis. Masson staining results revealed collagen fiber dissolution in the P group and DP group, along with muscle fiber dissolution and fragmentation specifically observed in the DP group (Fig. 9b). The extent of collagen fiber breakage was comparatively lower in the PL, LL-37, and PL/LL-37 groups. To assess the impact of PL, LL-37, and PL/LL-37 on osteoclasts, TRAP staining was performed as well (Fig. 9c). TRAP-positive cells exhibited characteristics of multinucleated giant cells with red-stained particles present in their cytoplasm. Notably, there was a significant increase in osteoclast numbers associated with enhanced bone destruction. In contrast to the P group and DP group where only a few osteoclasts were observed (Fig. 9c), no osteoclasts were detected in the treatment groups receiving PL, LL-37, or PL/LL-37. This suggests that these treatments effectively inhibit osteoclast formation within diabetic periodontitis periodontal tissues while also suppressing periodontal bone resorption.

#### 3.4.4 IHC

**Fig. 10:**
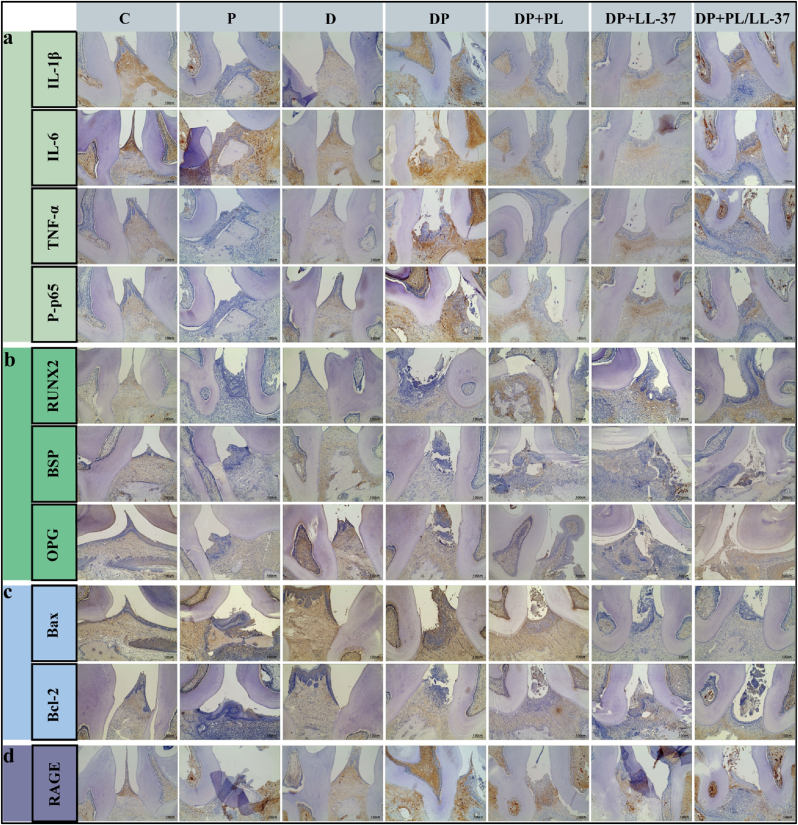
PL/LL-37 can exert anti-inflammatory, bone-enhancing, anti-apoptotic and diabetes-protective properties in diabetic periodontitis rats. (a) PL/LL-37 reduces inflammation levels in periodontal tissues of SD rats with diabetic periodontitis. Immunohistochemical staining of inflammation-related proteins (IL-1β, IL-6, TNF-α, NF-κB P-p65). (b) PL/LL-37 promotes bone regeneration in periodontal tissues of SD rats with diabetic periodontitis. Immunohistochemical staining of osteogenesis--related proteins (RUNX2, BSP, OPG). (c) PL/LL-37 exerts anti-apoptotic effects in SD rats with diabetic periodontitis. Immunohistochemical staining of Bax, and Bcl-2. (d) PL/LL-37 exerts diabetic protective effect in SD rats with diabetic periodontitis. Immunohistochemical staining of RAGE.

The positive cells exhibited a brown color in the IHC staining. IHC results of inflammation-related proteins (IL-1β, IL-6, TNF-α, and P-p65) are presented in Fig. 10a. Except for the P group and the DP group, no significant positive results were observed in all other groups. Therefore, PL, LL-37, and PL/LL-37 demonstrated their ability to attenuate inflammation levels in periodontal tissues of rats with diabetic periodontitis through modulation of the NF-κB P-p65 pathway. Although, regarding PL, LL-37, and PL/LL-37 regulation of macrophage differentiation, the results of this experiment in terms of PCR, WB, and IHC were obtained, and the results showed that PL/LL-37 was able to reduce the production of inflammatory factors and proteins in M1-type macrophages and promote the production of anti-inflammatory factors in M2-type macrophages, which confirms that PL/LL-37 can regulate the production of M1-type macrophages into M2-type macrophages, thus exerting anti-inflammatory effects. However, immunostaining for specific proteins of M1-type and M2-type macrophages was lacking to further confirm the effects of PL, LL-37 and PL/LL-37 on macrophage polarization. This we will continue to study in depth in the future.

IHC results of osteogenesis-related proteins (RUNX2, BSP, and OPG) are shown in Fig. 10b. Positive cell staining was observed in the PL, LL-37, and PL/LL-37 groups while no significant positive results were found in the remaining groups. This indicates that PL, LL-37, and PL/LL-37 promote bone regeneration within periodontal tissues. The IHC results of apoptosis-related proteins, Bcl-2 and Bax, are presented in Fig. 10c. Regarding Bax, positive cells with a darker staining background were observed in all groups except for the PL/LL-37 group, particularly prominent in the DP group. Concerning Bcl-2, positive cells were detected in the PL, LL-37, and PL/LL-37 groups; however, no significant positivity was observed in the other groups. Henceforth, PL, LL-37, and PL/LL-37 exhibited anti-apoptotic effects by downregulating Bax expression while upregulating Bcl-2 production. IHC results of RAGE (Fig. 10d) revealed significant positive outcomes only in the P, D, and DP groups, while no other groups exhibited noteworthy effects. Consequently, PL, LL-37, and PL/LL-37 may confer a protective effect against diabetes by mitigating ligand response of the AGE-RAGE receptor and ameliorating periodontal tissue damage induced by inflammation caused by AGE accumulation, oxidative stress, and PMN respiratory burst.

Altered osteogenic-osteoclastic balance within the alveolar bone is a crucial mechanism in the pathogenesis of periodontal disease in individuals with diabetes, involving nuclear factor receptor activator-κB ligand/OPG (RANKL/OPG) [64]. RANKL stimulates osteoclastogenesis and activation by binding to its receptor RANK, while OPG inhibits this pro-osteoclastogenic effect by competing with RANKL for the RANK binding site [65]. Poor glycemic control adversely affects the RANKL/OPG ratio in diabetic patients, leading to increased formation of osteoclasts [66]. The treatment of periodontitis in diabetic patients relies on alveolar bone regeneration, which is influenced by Runx2, an osteogenic transcription factor that regulates multiple factors' transcription and extracellular matrix protein secretion, thereby promoting osteogenic differentiation and inhibiting osteoclast formation [67]. BSP, a phosphorylated and sulphated glycoprotein, serves as the principal non-collagenous protein in bone and other mineralized connective tissues, playing a crucial role in regulating the initial stages of connective tissue mineralization with specific involvement [68]. In this study, PL/LL-37 demonstrated inhibitory effects on osteoclastogenesis while promoting the expression of osteogenesis-related proteins (RUNX2, BSP, and OPG), thereby exerting regulatory functions on bone metabolism and facilitating periodontal tissue regeneration in diabetic periodontitis.

Apoptosis, also known as programmed cell death, is carried out at all stages of organism growth and development by mitochondrial-mediated endogenous apoptosis, in addition to exogenous apoptosis, which is mainly controlled by the Bcl-2 family of proteins [69]. The Bcl-2 family of proteins is an evolutionarily conserved molecule, which plays a key role in controlling apoptotic protein release from mitochondria, and promotes or inhibits apoptosis by regulating mitochondrial structure and function to play a role in promoting or inhibiting apoptosis [70]. Among them, Bcl-2 is a typical anti-apoptotic gene, while Bax is a typical representative of pro-apoptotic genes [69]. In this study, we used IHC to observe the expression of Bax and Bcl-2 in the periodontal tissues of rats in various groups. We found that the expression of Bax was significantly higher in the P, D, and DP groups compared to the DP + PL, DP + LL-37, and DP + PL/LL-37 groups. Additionally, Bcl-2 was expressed in the periodontal tissues of rats in the DP + PL, DP + LL-37, and DP + PL/LL-37 groups. This suggests that PL/LL-37 could upregulate the expression of Bcl-2 while downregulating the expression level of Bax, thereby resisting apoptosis occurrence in rat periodontal tissues with diabetic periodontitis.

During glycolysis, glucose metabolism generates methylglyoxal, a carbonyl intermediate, and forms a series of irreversibly glycosylated proteins and lipoproteins known as AGE, which can contribute to IR and hyperglycemia [71]. AGEs exert their effects by binding to the transmembrane protein receptor for AGEs (RAGE) Compared to non-diabetic patients with chronic periodontitis, individuals with diabetic periodontitis exhibit significantly elevated levels of RAGE [72]. Previous studies have suggested that the accumulation of AGEs and alterations in its receptor RAGE in periodontal tissues may be responsible for increased periodontal destruction in patients with diabetic periodontitis [18]. Inflammation and oxidative stress induced by interactions between AGEs and RAGE in periodontal tissues promote monocyte/macrophage activation as well as endothelial cell release of pro-inflammatory cytokines such as IL-1, IL-6, and TNF-α, ultimately leading to disruption of the RANKL/OPG axis and subsequent increase in bone resorption [18]. In this study, PL/LL-37 exhibited the ability to downregulate RAGE levels in periodontal tissues affected by diabetic periodontitis (Fig. 10d), thereby mitigating the AGE-RAGE response and subsequent damage to periodontal tissues, thus demonstrating its therapeutic potential for treating diabetic periodontitis.

### 3.5 Mechanism of action of PL/LL-37 in the treatment of diabetic periodontitis

Due to the diverse pathogenesis of diabetic periodontitis, a comprehensive treatment approach is essential to mitigate its adverse effects. It remains a challenge to identify anti-diabetic periodontitis agents that possess multiple biological activities. The combination of PL/LL-37 could exert antibacterial, anti-inflammatory, antioxidant, diabetes-protective effects, promote bone health, and regulate apoptosis, rather than relying solely on a simple anti-inflammatory and antibacterial strategy. Furthermore, compared to LL-37 alone, the combination of PL/LL-37 exhibited enhanced structural stability and safety profiles while also providing superior antibacterial activity as well as improved anti-inflammatory, antioxidant and osteogenic effects alongside diabetes protection.

**Fig. 11:**
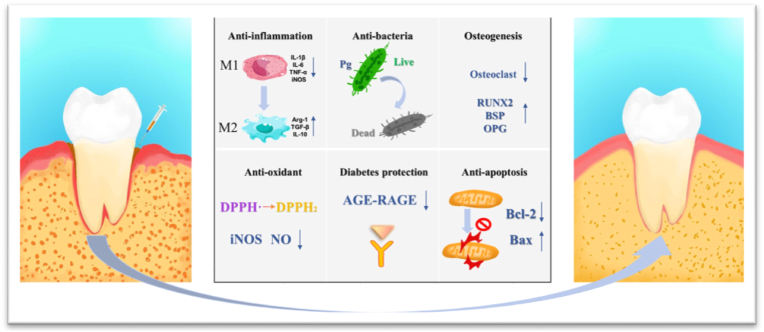
Schematic illustration of the mechanism of the PL/LL-37 in diabetic periodontitis treatment.

PL/LL-37 possesses the advantages of multi-functionality and low toxicity, demonstrating favorable properties in terms of antibacterial activity, anti-inflammatory response, antioxidant capacity, osteogenesis promotion, anti-apoptotic effects, and diabetes protection within the microenvironment of diabetic periodontitis. Consequently, it facilitates the transition of periodontal tissues affected by diabetic periodontitis from an exacerbated high-glycemic inflammatory state to a regenerative microenvironment (Fig. 11). The exceptional performance exhibited by PL/LL-37 in promoting regeneration of diabetic periodontal tissues can be attributed to the following factors. First, PL/LL-37 exhibited potent antimicrobial properties. It effectively inhibited the adhesion, growth, and biofilm formation of *P. gingivalis* while also disrupting pre-formed biofilms. The underlying mechanism of its antimicrobial activity involved the formation of ion channels on microbial cell membranes and binding to bacterial membrane endotoxins. Second, PL/LL-37 demonstrated remarkable anti-inflammatory properties. By modulating macrophage polarization from an M1 phenotype to an M2 phenotype through the NF-κB P-p65 inflammatory pathway, it significantly reduced the release of pro-inflammatory cytokines and promoted the secretion of anti-inflammatory cytokines. Third, PL/LL-37 displayed excellent antioxidant properties by scavenging DPPH free radicals and inhibiting the production of iNOS and NO. Fourth, PL/LL-37 exhibited osteogenic properties by promoting the production of RUNX2, BSP, and OPG osteogenic proteins while modulating the RANKL-OPG axis to decrease osteoclastogenesis, thereby facilitating bone regeneration in diabetic periodontitis. Fifth, PL/LL-37 demonstrated significant anti-apoptotic effects by downregulating Bax expression and upregulating Bcl-2 expression. Sixth, PL/LL-37 exerted a protective effect against diabetes by reducing the accumulation of RAGE in periodontal tissues and subsequently attenuating the AGE-RAGE response.

## 4 Conclusions

Targeting the key aspects of diabetic periodontitis treatment, we developed a multi-bioactive PL/LL-37 with anti-periodontal pathogen, antioxidant, immune regulatory, diabetic protective, and osteogenic properties. Firstly, our study demonstrates that the incorporation of PL alters the secondary conformation of LL-37, rendering PL/LL-37 more stable and antimicrobial compared to LL-37 alone. Moreover, PL/LL-37 not only inhibits the growth and adhesion of *P. gingivalis* but also disrupts mature biofilms, thereby impeding plaque formation and reducing precursors associated with periodontitis development. Secondly, in the hyperglycemic periodontitis microenvironment excessive immune responses can be modulated through downregulation of pro-inflammatory cytokines and upregulation of anti-inflammatory cytokines via NF-kB p-P65 signaling pathway as well as other pathways. Thirdly, it is postulated that PL/LL-37 may exert a protective role in diabetes and mitigate periodontal tissue damage by means of its antioxidant properties, as well as through the reduction of AGE-RAGE reaction triggered by AGE accumulation. Moreover, due to its anti-periodontal pathogen activity, antioxidant potential, immune regulatory effects, diabetic protection capabilities, and osteogenesis-inducing properties, PL/LL-37 demonstrates promising therapeutic efficacy in SD rats with diabetic periodontitis. Compared to LL-37 alone, PL/LL-37 exhibits enhanced structural stability and safety profile while displaying superior antibacterial, anti-inflammatory, antioxidant and diabetes protective effects. These findings highlight the diverse biological activities of PL/LL-37 and underscore its broad clinical application prospects for the treatment of diabetic periodontitis.

## CRediT authorship contribution statement

**Cancan Li:** Writing – original draft, Validation, Software, Methodology, Formal analysis, Data curation, Conceptualization. **Luowen Du:** Methodology, Investigation, Formal analysis, Data curation. **Yingying Xiao:** Validation, Methodology, Investigation. **Lei Fan:** Validation, Methodology, Investigation. **Quanli Li:** Supervision, Resources, Project administration, Funding acquisition, Conceptualization. **Chris Ying Cao:** Writing – review & editing, Supervision, Resources, Project administration, Funding acquisition, Conceptualization.

## Ethics approval and consent to participate

All rat experiments were approved by the Animal Ethics Committee of Anhui Medical University under ethical number LLSC20232193.

## Declaration of competing interest

The authors declare that they have no known competing financial interests or personal relationships that could have appeared to influence the work reported in this paper.

## Data Availability

Data will be made available on request.
