# Supplementary material for: Multi-active phlorotannins boost antimicrobial peptide LL-37 to promote periodontal tissue regeneration in diabetic periodontitis
Source: Mater Today Bio. 2025 Jan 31;31:101535. doi: 10.1016/j.mtbio.2025.101535 (PMC11847560; doi:10.1016/j.mtbio.2025.101535)
Supplement: Multimedia component 1 [file mmc1.docx]

**Multi-active Phlorotannins Boost Antimicrobial Peptide LL-37 to Promote Periodontal Tissue Regeneration in Diabetic Periodontitis**

Cancan Li^a^, Luowen Du^a^, Yingying Xiao^a^, Lei Fan^a^, Quanli Li^b^, Chris Ying Cao^a,^ *

^a^ College & Hospital of Stomatology, Anhui Medical University, Key Lab. of Oral Diseases Research of Anhui Province, Hefei, 230032, China

^b^ Institute of Oral Science, Department of Stomatology, Longgang Otorhinolaryngology Hospital of Shenzhen, Shenzhen, 518172, China

*Corresponding author

E-mail address: caoying0713@gmail.com (Chris Ying Cao)

Table S1. Primer sequence of inflammation-related genes

| Gene | Forward (5′-3′) | Reverse (5′-3′) |
| --- | --- | --- |
| β-actin | CATCCGTAAAGACCTCTAGCCAAC | ATGGAGCCACCGATCCACA |
| TNF-α | ACTCCAGGCGGTGCCTATGT | GTGAGGGTCTGGGCCATAGAA |
| IL-6 | CCACTTCACAAGTCGGAGGCTTA | CCAGTTTGGTAGCATCCATCATTTC |
| IL-1β | TCCAGGATGAGGACATGAGCAC | GAACGTCACACACCAGCAGGTTA |
| iNOS | ATCTTGGAGCGAGTTGTGGATTGTC | TAGGTGAGGGCTTGGCTGAGTG |
| IL-10 | ATGCTGCCTGCTCTTACTGACTG | CCCAAGTAACCCTTAAAGTCCTGC |
| Arg-1 | CTCCAAGCCAAAGTCCTTAGAG | AGGAGCTGTCATTAGGGACATC |
| TGF-β | GAAGAGGGTGGAAGCCATTAG | GAGGACACATTGAAACGGAAA |


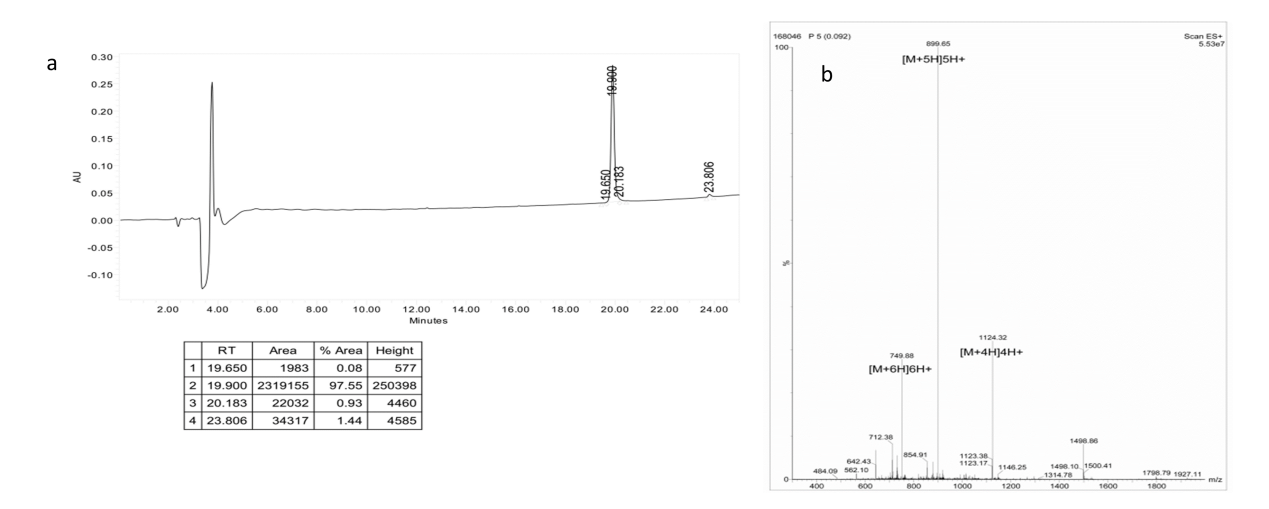


Figure S1. Identification of Antimicrobial peptides LL-37. (a) HPLC chromatogram at 220 nm. (b) MS spectrum.


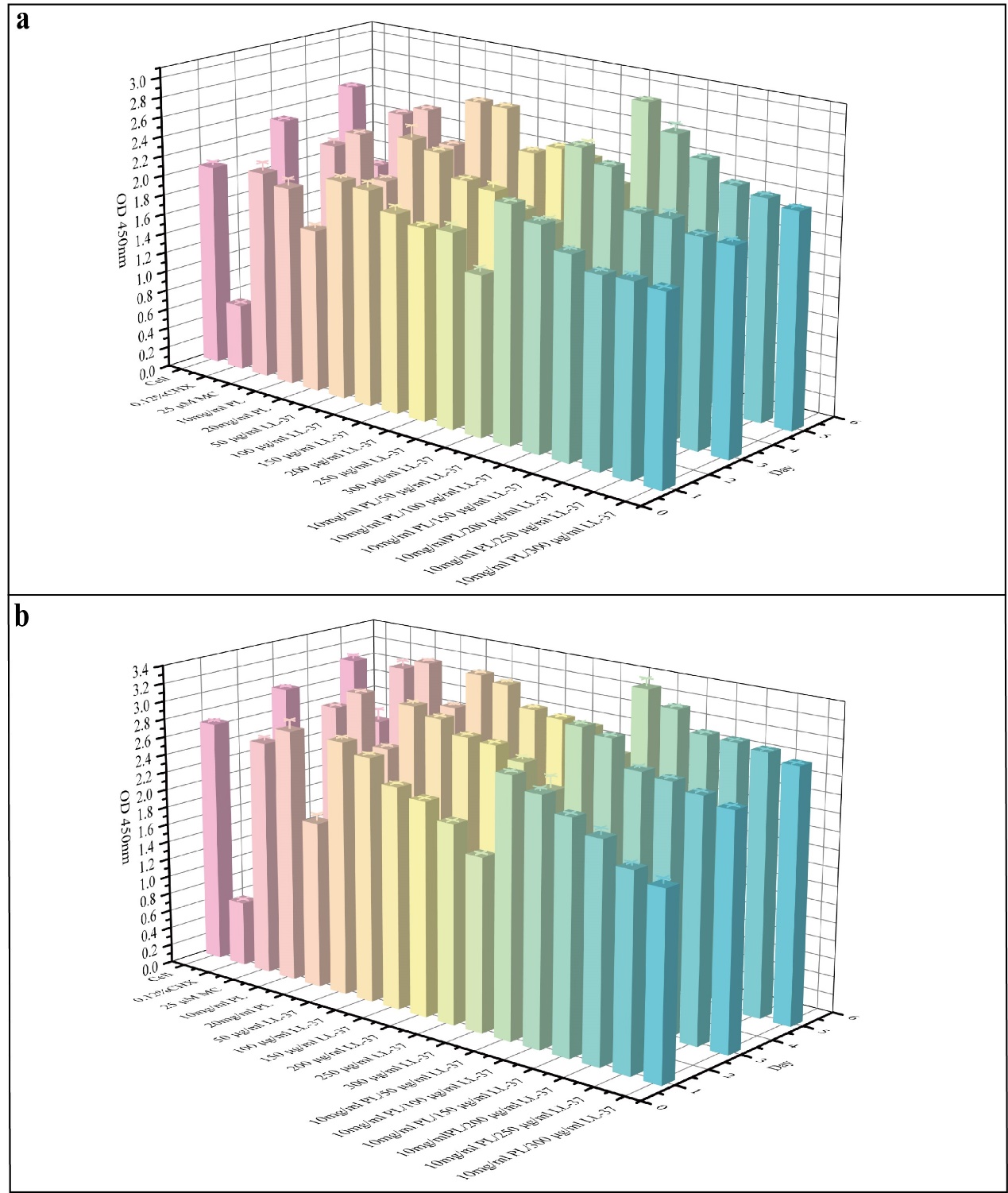


Figure S2. CCK-8 assay in BMSCs and RAW 264.7 cells under different concentrations of PL, LL-37 and PL/LL-37 over time. (a) BMSCs. (b) RAW 264.7 cells.


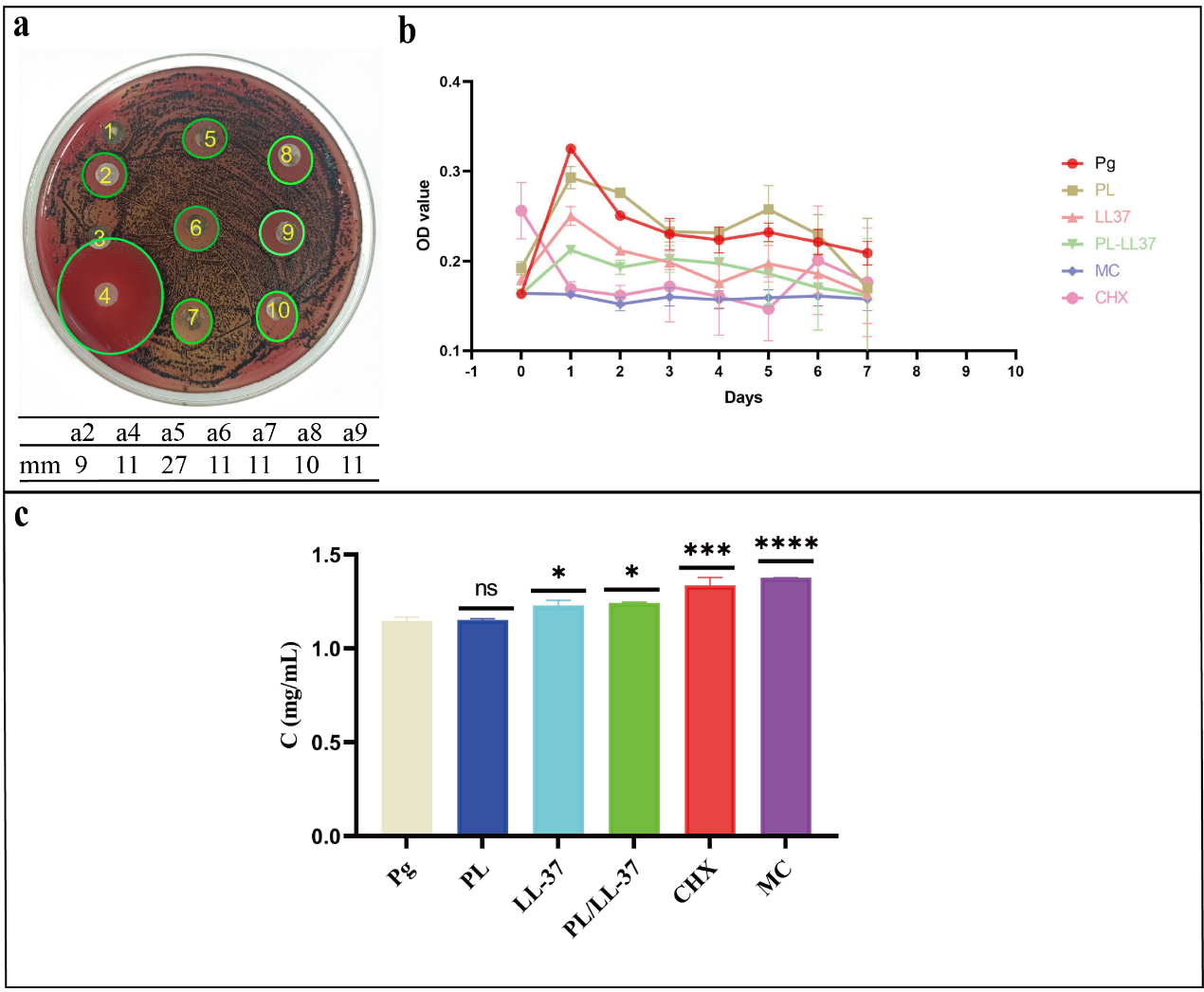


Figure S3. The antibiofouling abilities of PL, LL-37 and PL/LL-37. (a) Disc Diffusion. (1) 10 mg/mL PL. (2) 100 μg/mL LL-37. (3) PBS. (4) 0.12% CHX. (5) PL/LL-37 (10 mg/mL PL, 50 μg/mL LL-37). (6) PL/LL-37 (10 mg/mL PL, 100 μg/mL LL-37). (7) PL/LL-37 (10 mg/mL PL, 150 μg/mL LL-37). (8) PL/LL-37 (10 mg/mL PL, 200 μg/mL LL-37). (9) PL/LL-37 (10 mg/mL PL, 250 μg/mL LL-37). (10) PL/LL-37 (10 mg/mL PL, 300 μg/mL LL-37). (b)OD monitoring curve for inhibition of *P. gingivalis* growth. (c) The BCA assay to assess bacterial lysis.


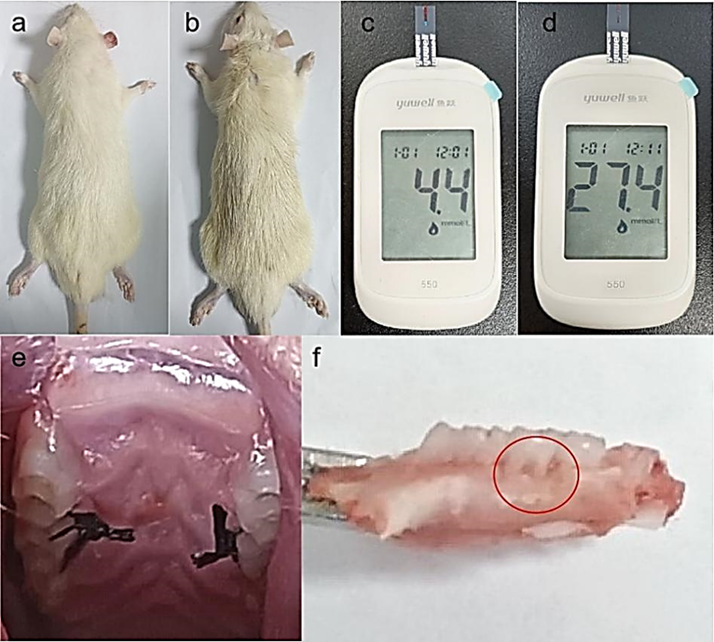


Figure S4. The successful modelling of diabetic periodontitis SD rats. (a) Hair of non-diabetic SD rats. (b) Hair of diabetic SD rats. (c) Random blood glucose of non-diabetic SD rats. (d) Random blood glucose of diabetic SD rats. (e) Silk wire ligation at the cervical region of maxillary second molar in SD rats. (f) Successful periodontitis modelling.
